# Ga(III) pyridinecarboxylate complexes: potential analogues of the second generation of therapeutic Ga(III) complexes?

**DOI:** 10.1007/s00775-023-02012-2

**Published:** 2023-07-27

**Authors:** Michaela Rendošová, Róbert Gyepes, Simona Sovová, Danica Sabolová, Mária Vilková, Petra Olejníková, Martin Kello, Boris Lakatoš, Zuzana Vargová

**Affiliations:** 1 Department of Inorganic Chemistry, P. J. Šafárik University, Moyzesova 11, 041 54 Kosice, Slovak Republic; 2 Department of Inorganic Chemistry, Charles University, Hlavova 2030, 128 00 Prague, Czech Republic; 3 Department of Biochemistry, P. J. Šafárik University, Moyzesova 11, 041 54 Kosice, Slovak Republic; 4 NMR Laboratory, P. J. Šafárik University, Moyzesova 11, 041 54 Kosice, Slovak Republic; 5 Department of Biochemistry and Microbiology, Slovak University of Technology, Radlinského 9, 812 37 Bratislava, Slovak Republic; 6 Department of Pharmacology, P. J. Šafárik University, Trieda SNP 1, 040 11 Kosice, Slovak Republic

**Keywords:** Ga(III) complexes, Anticancer, Antimicrobial, BSA binding, Stability, Potentiometry

## Abstract

A series of novel Ga(III)—pyridine carboxylates ([Ga(Pic)_3_]·H_2_O (GaPic; HPic = picolinic acid), H_3_O[Ga(Dpic)_2_]·H_2_O (GaDpic; H_2_Dpic = dipicolinic acid), [Ga(Chel)(H_2_O)(OH)]_2_·4H_2_O (GaChel; H_2_Chel = chelidamic acid) and [Ga(Cldpic)(H_2_O)(OH)]_2_ (GaCldpic; H_2_Cldpic = 4-chlorodipicolinic acid)) have been synthesized by simple one-step procedure. Vibrational spectroscopy (mid-IR), elemental analysis, thermogravimetric analysis and X-ray diffraction confirmed complexes molecular structure, inter and intramolecular interactions and their influence to spectral and thermal properties. Moreover, complex species speciation was described in Ga(III)-HPic and Ga(III)-H_2_Dpic systems by potentiometry and ^1^H NMR spectroscopy and mononuclear complex species were determined; [Ga(Pic)_2_]^+^ (log*β*_*021*_ = 16.23(6)), [Ga(Pic)_3_] (log*β*_*031*_ = 20.86(2)), [Ga(Dpic)_2_]^−^ (log*β*_*021*_ = 15.42(9)) and [Ga(Dpic)_2_(OH)]^2−^ (log*β*_*-121*_ = 11.08(4)). To confirm the complexes stability in 1% DMSO (primary solvent for biological testing), timescale ^1^H NMR spectra were measured (immediately after dissolution up to 96 h). Antimicrobial activity evaluated by IC_50_ (0.05 mM) is significant for GaDpic and GaCldpic against difficult to treat and multi-resistant *P. aeruginosa*. On the other hand, the GaPic complex is most effective against Jurkat, MDA-MB-231 and A2058 cancer cell lines and significantly also decreases the HepG2 cancer cells viability at 75 and 100 μM concentrations in a relatively short time (up to 48 h). In addition, fluorescence measurements have been used to elucidate bovine serum albumin binding activity between ligands, Ga(III) complexes and bovine serum albumin.

**Graphical abstract:**

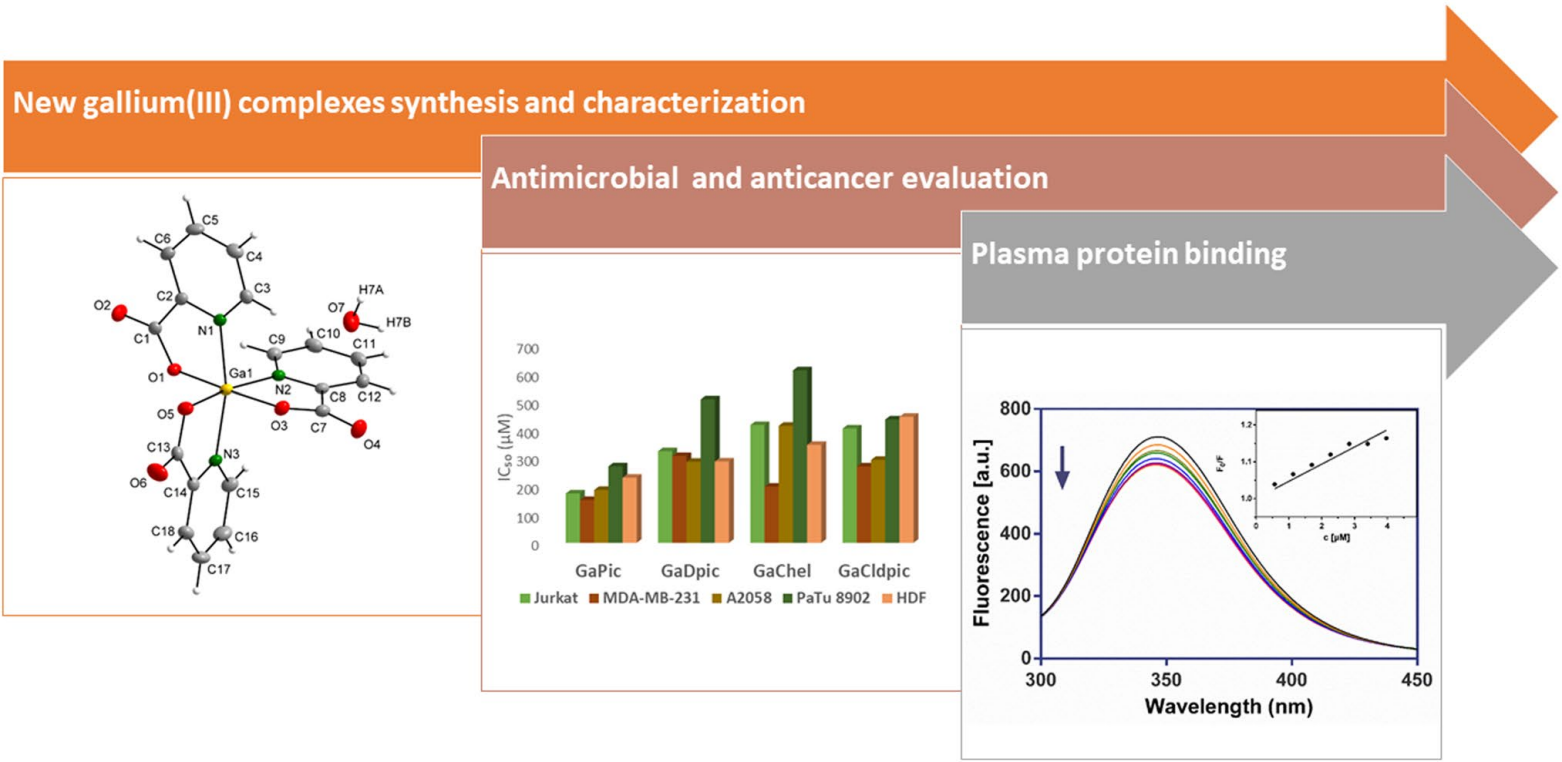

**Supplementary Information:**

The online version contains supplementary material available at 10.1007/s00775-023-02012-2.

## Introduction

The resistance of various microbes in recent years leads to the development of diseases that are problematic to treat with currently available antibiotics [1]. Among the best known are the developed resistance of *S. pneumoniae* to penicillin and other antibiotics, *Staphylococcus aureus* to methicillin, *Mycobacterium tuberculosis* to antituberculotics, *Neisseria gonorrhoeae* to beta-lactams and quinolones, *Escherichia coli* to fluorinated quinolones, cephalosporins, aminopenicillins and clotrimoxazole. Unlike classical antibiotics, which target a specific reaction or process, metals, metal ion coordination complexes and metallo-organic compounds often affect several different groups of biomolecules, and therefore, development of resistance is unlikely, which makes these compounds attractive as antibacterial agents [2]. Their wide range of oxidation states, structures, geometries, solubilities, rates of ligand exchange, strengths of metal–ligand bonds, Lewis acidity, metal- and metal–ligand redox potentials offer new suitable entities with various attractive biological properties, such as antimicrobial but also anticancer. For antimicrobial applications, the most studied metal compounds today are silver(I), copper(II), zinc(II), ruthenium(II) and gallium(III), bismuth(III), indium(III), iron(III), vanadium(V) and others [3]. In general, current knowledge about the mechanisms of action of metal ion compounds against pathogenic microorganisms or cancer cell lines can be summarized in several ways, such as: exchange or release of ligands, redox activation and catalytic generation of toxic species (reactive oxygen species, ROS), as well as depletion of essential substrates [4, 5], making them able to abolish enzyme activities, disrupt membrane function or damage DNA.

In our previous work in the case of silver(I) and zinc(II) complexes with various predominantly biocompatible ligands (e.g. amino acids, dipeptides, pyridine carboxylates) we also found a significant biological effect of silver(I) complexes, mainly against bacteria and also against various cancer cell lines [6–9]. Our results indicate their activity mechanism in bacteria membrane disruption and DNA molecule damage.

Due to the latest knowledge from the perspective of metal ion compounds for antimicrobial and anticancer treatment, gallium(III) compounds have also been studied intensively in recent years. The compounds Ga(III) maltolate (GaM) and Ga(III) tris(8-quinolinolato) (KP46) as 2nd generation of therapeutic Ga(III) complexes appear to be the most suitable because gallium(III) nitrate causes high nephrotoxicity and complicated in vivo speciation (low effective concentration) [10]. Maltolate and 8-quinolinolato bidentate and chelate ligands adjust the Ga(III) speciation in vivo and increase its activity against cells. Although the effect of Ga(III) complexes is associated with damage to Fe-dependent processes, e.g., inhibition of Fe-containing proteins in the respiratory chain, Keppler et al. demonstrated that KP46 kills cancer cells mainly through upregulating the p53 and leading to increase in cellular Ca(II) and ROS level [11]. The DNA damage induced by KP46 was attributed to the increasing oxidative stress rather than direct interaction KP46 and DNA in a manner of cisplatin. Moreover, the complexation of Ga(III) with tridentate chelators like thiosemicarbazone enhances the biological activity of Ga(III) and improves pharmaceutical properties, so they are considered to be an important class of cytotoxic drugs with different antiproliferative mechanisms [12–14]. In addition, the inhibition properties of Ga(III) mixed ligand complexes (used ligands = 2-aminobenzimidazole with pyridine-2,6-dicarboxylic acid and 4-hydroxypyridine-2,6-dicarboxylic acid) were studied in vitro using oxaliplatin as a standard against five cell cancer lines and the results showed MCF7 cells were the most sensitive cell line to cytotoxicity of the complexes [15]

To determine whether gallium(III) picolinato and dipicolinato complexes with a similar structure as gallium(III) quinolinolato, maltolato and thiosemicarbazone complexes have such excellent biological properties, we prepared, characterized and biologically tested them. In addition, we monitored their stability in testing biological media and described their speciation in aqueous solution in order to know what complexing species can be expected in solution at different pH values. To complete their biological testing, BSA interactions with selected complexes were observed.

As gallium is known to accumulate in hepatocellular tumours (based on ^67^ Ga scans) [16, 17] and there is a compelling rationale for investigating the potential utility of gallium in the treatment of hepatocellular carcinoma, the work is enriched with a chapter on the cytostatic effect of the new Ga(III) picolinate complex against HepG2 (human liver cancer cell line), which is compared to its Ag(I) and Zn(II) analogues effect.

## Experimental

### Materials and methods

Picolinic, dipicolinic, and chelidamic acid, dimethyl sulfoxide (DMSO) and bovine serum albumin (BSA) were received from Sigma-Aldrich Chemicals. 4-chlorodipicolinic acid was prepared according to literature procedure [18].

Gallium(III) nitrate hydrate was purchased from Alfa Aesar and was dried under vacuum at 60 °C. Other chemicals were analytically pure and used without purification.

## Solid state study

### GaPic, GaDpic, GaChel, GaCldpic complexes syntheses

#### Synthesis of [Ga(Pic)_*3*_]·H_2_*O (GaPic)*

A solution of gallium(III) nitrate (100 mg; 0.39 mmol) in water (5 ml) was slowly added to a 5 ml water solution of HPic (96 mg; 0.78 mmol) heated to 50 °C. The reaction mixture was stirred for 30 min and then was kept for slow evaporation. Transparent crystals formed after 2 weeks were filtered off, dried with diethyl ether and were used for further characterization. Yield: 48%. Anal. calcd. for C_18_H_14_Ga_1_N_3_O_7_ (%): C 47.62; H 3.11; N 9.25; Found: C 47.41; H 3.11; N 9.24. MS( +) m/z: [Ga(Pic)_3_(H_2_O) + H]^+^ 457.9.

#### Synthesis of H_3_O[Ga(Dpic)_2_]·H_2_O (GaDpic)

A solution of gallium(III) nitrate (100 mg; 0.39 mmol) in water (5 ml) was slowly added to a 15 ml water solution of H_2_Dpic (131 mg; 0.78 mmol) heated to 50 °C. The reaction mixture was stirred for 30 min and then was kept for slow evaporation. Transparent crystals formed after 2 weeks were filtered off, dried with diethyl ether and then were used for further characterization. Yield: 43%. Anal. calcd. for C_14_H_11_Ga_1_N_2_O_10_ (%): C 38.48; H 2.54; N 6.41; Found: C 37.61; H 2.37; N 6.16. MS(-) m/z: [Ga(Dpic)_2_]^−^ 399.1.

#### Synthesis of [Ga(Chel)(H_2_O)(OH)]_2_·4H_2_O (GaChel) and [Ga(Cldpic)(H_2_O)(OH)]_2_ (GaCldpic)

5 mL of aqueous gallium(III) nitrate solution (100 mg; 0.39 mmol) was added dropwise to the solution of chelidamic acid (72 mg; 0.39 mmol) / 4-chlorodipicolinic acid (79 mg; 0.39 mmol) ethanol:water mixture (40 ml; 1:3) heated to 50 °C. The reaction mixture was stirred for 30 min, filtered and then allowed to crystallize. After 10 days light brown crystals of GaChel / yellowish crystals of GaCldpic were isolated by filtration, washed with diethyl ether and air-dried. Yield (for GaChel): 42%. Anal. calcd. for C_14_H_20_Ga_2_N_2_O_18_ (GaChel; %): C 26.12; H 3.13; N 4.35; Found: C 26.51; H 3.09; N 4.11. MS(-) m/z: [Ga(Chel)_2_]^−^ 431.0. Yield (for GaCldpic): 51%. Anal. calcd. for C_14_H_10_Cl_2_Ga_2_N_2_O_12_ (GaCldpic; %): C 27.63; H 1.66; N 4.60; Found: C 27.62; H 1.87; N 4.22. MS( +) m/z: [Ga(Cldpic-OCO)_2_]^+^ 381.2.

### Physical measurements

*Single-crystal diffraction* data for complexes GaDpic, GaChel and GaCldpic were collected on a Bruker D8 VENTURE Kappa Duo PHOTON 100 diffractometer using Mo Kα radiation for GaDpic and GaCldpic (λ = 0.71073 Å) and Cu Kα radiation for GaChel (λ = 1.54178 Å). Crystal structure of GaPic complex was measured with Nonius Kappa CCD (charge-coupled device) diffractometer equipped with a Bruker APEX II detector and graphite monochromator, using Mo Kα radiation (λ = 0.71073 Å). The structures were solved by direct methods using SHELXT [19] and refined on F^2^ using SHELX [20]. All non-hydrogen atoms were refined anisotropically and aromatic hydrogens were included in the calculated positions. The intermolecular interactions were analysed by PLATON [21]. Crystal data for complexes are listed in Table S1. The structure figures were drawn using the DIAMOND software [22].

*Infrared spectra* of the compounds were recorded on Avatar FT-IR 6700 (Fourier transform infrared spectroscopy) spectrometer from 4000 to 400 cm^−1^ using ATR (attenuated total reflectance) technique.

*Elemental analysis* was performed with a CHNOS Elemental Analyzer Vario MICRO from Elementar Analysensysteme GmbH.

*Thermal behaviour* of compounds GaPic and GaDpic was studied by thermogravimetry (TG) using TA instrument TGA Q-500 and of GaChel and GaCldpic complexes using Setaram Setsys Evolution analyser-1750 in the atmosphere of air. Samples were heated with a heating rate of 10 °C.min^−1^ in the temperature range from 25 to 800 °C (for complexes GaPic and GaDpic)/ 700 °C (for complexes GaChel and GaCldpic) and air flow rate 60 cm^3^.min^−1^. Before thermal measurements, gentle grinding of the sample and careful packing into the platinum crucibles were performed. The mass of samples used in the analyses was within 5–20 mg. Obtained thermoanalytical curves were analysed using Origin computational program (version b9.3.226, OriginLab Corp., Northampton, MA, USA, 2016).

*Mass spectra* were recorded on Bruker ESQUIRE 3000 ion-trap spectrometer with electrospray ionization in negative or positive mode in the range of 50 to 1000 m/z and the most abundant ion is presented. The compound GaChel was dissolved in methanol and GaPic, GaDpic, GaCldpic in acetonitrile of HPLC quality.

### Complexes stability study in biological testing medium

Nuclear magnetic resonance (NMR) data were collected on a Varian VNMRS 600 spectrometer operating at 599.87 MHz for ^1^H. The concentration of all samples was approximately 3 mg/0.6 mL of 1% DMSO-d_6_/D_2_O. For comparison, the spectra of pure acids were also measured, while the pH of the solution of acids was adjusted to the same value as the solution of the complexes. The chemical shifts were referenced to the TSP (3-(trimethylsilyl)propionic-2,2,3,3-d_4_ acid sodium salt) peak (^1^H NMR 0.00 ppm). The NMR data were recorded at 300 K, with chemical shifts *δ* reported in parts per million and coupling constants J in Hertz. All data were analysed using MestReNova 12.0.0 (2017) software. The stability in 1% DMSO-d_6_/D_2_O was determined for all samples. Stability under these conditions is a prerequisite for biological studies and it was determined by ^1^H NMR spectroscopy over a time span of 5 days.

## Solution measurements

### Potentiometry

The stock solution of HPic and H_2_Dpic was prepared by dissolving in demineralized water (*c*(ligand) = 0.02 M). The gallium(III) ion solution was prepared from anhydrous gallium(III) nitrate (*c*(Ga^3+^) = 0.05 M) and was standardized by back titration of excess of EDTA with zinc(II). Decarbonated potassium hydroxide solution (approx. 0.2 M) was used as a titrant that was standardized by potassium hydrogen phthalate (*c*(KHP) = 0.1 M). Standard inert electrolyte of nitric acid was used in all titrations (*c*(HNO_3_) = 0.03 M, *I*(HNO_3_/KNO_3_) = 0.1 M). Potentiometric measurements were carried out using a TitroLine 7000 dosage system with a BlueLine combination electrode (SI Analytics) in a glass vessel (5 mL) thermostated at 25 ± 0.1 °C at an ionic strength *I*(KNO_3_) = 0.1 M. Inert atmosphere was ensured by a constant flow of a nitrogen gas. Precise calibration of the electrode was carried out by the titration of 0.03 M HNO_3_ with 0.2 M KOH in the range –log[H^+^] = 1.8–12.0, with the pH-meter yielding E values. The relation between E and –log[H^+^] is expressed by Eq. (1) where the term E^0^ contains the standard potentials of the electrode and the contribution of inert ions to the liquid-junction potential. The value S corresponds to the Nernstian slope, the value of which should be close to the theoretical value, and *j*_*1*_[H^+^] and *j*_*2*_[OH^–^] = *j*_*2*_*K*_*w*_/[H^+^] are contributions of the H^+^ and OH^–^ ions, respectively, to the liquid-junction potential. The p*K*_*w*_ value was 13.78 in Eq. (1).

1 $$E \, = \, E^{0} {-} \, S\left( {{-}log\left[ {H^{ + } } \right]} \right) \, + \, j_{1} \left[ {H^{ + } } \right] \, + \, j_{2} K_{w} /\left[ {H^{ + } } \right]$$

The parameters j_1_ and j_2_ cause deviation from linear dependence between E and –log[H^+^] only in strongly acidic and strongly alkaline solutions. The picolinic and dipicolinic acid protonation constants were determined with ligand concentration 0.004 M. The stability constants in the Ga-HPic and Ga-H_2_Dpic binary system were determined by the ligand concentrations 0.004 and the Ga^3+^ concentration was 0.004, 0.002 and 0.001 M in dependence on the ligand to metal ratio.

The protonation constants *β*_110_, *β*_210_ are the concentration constants and are defined by *β*_110_ = [HL]/ [H][L], *β*_210_ = [H_2_L]/ [H]^2^[L] (p*K*_2_ = log*β*_110_, p*K*_1_ = log*β*_210_- log*β*_110_); the stability constants are defined by *β*_rqp_ = [H_r_L_q_M_p_]/[H]^r^[L]^q^[M]^p^. The equilibrium constants were obtained by fitting the titration data with OPIUM [23]. The following hydrolysis constants were used for the calculations: *β*_pr_ = [M_p_(OH)_r_^3−r^][H]^r^/[M]^p^; log *β*_11_ = − 2.74; log *β*_12_ = − 7.0; log *β*_13_ = − 11.96; log *β*_14_ = − 15.52 [24].

### ^1^H NMR titrations

^1^H NMR spectra were recorded on Varian VNMRS 600 MHz spectrometer (^1^H-599.87 MHz). Samples were prepared in non-deuterated water and measured with D_2_O insert containing TSP (3-(trimethylsilyl)propionic-2,2,3,3-d_4_ acid sodium salt, 0.00 ppm) used for reference. Four FIDs were accumulated for each spectrum using standard transmitter presaturated pulse sequence.

The initial volume of solutions for each titration point was 2 cm^3^. In the titration experiments, solutions contained aliquots of Ga(NO_3_)_3_ and acid HPic or H_2_Dpic. The initial concentration of individual species was 0.04 M. Solutions pH values were adjusted by HNO_3_ solution (0.1 M) and KOH solution (0.1 M).

## Biological tests

### Antimicrobial activity

The antimicrobial activities of gallium(III) complexes GaPic, GaDpic, GaChel, GaCldpic, free ligands (HPic, H_2_Dpic, H_2_Chel, H_2_Cldpic) and positive controls (cefoxitin (C), ampicillin (A) and piperacillin (P)) were evaluated by macrodilution method [25, 26] using G^+^ bacteria (*Firmicutes*) *Staphylococcus aureus* (*S. aureus*) CCM 3953 (Czech Collection of Microorganisms), G^−^ bacteria (*γ-proteobacteria*) *Escherichia coli* (*E. coli*) CCM 3988, *Pseudomonas aeruginosa* (*P. aeruginosa*) CCM 1959 (Czech Collection of Microorganisms) and the yeast *Candida parapsilosis* (*C. parapsilosis*) ATCC 22019 (American type culture collection). Cultures of bacteria (in Mueller–Hinton broth, MHB) and yeasts (in Sabouraud’s growth medium, SB) were incubated under shaking (250 rpm) at 37 °C. The growth of bacteria and yeasts was evaluated by measuring the absorbance of the growing cultures (A590) until the cultures reached the stationary growth phase. The effects of gallium(III) complexes on the growth of filamentous fungi *Rhizopus oryzae* (*R. oryzae*) CCM F-8284, *Alternaria alternata* (*A. alternata*) CCM F-128 and *Microsporum gypseum* (*M. gypseum*) CCM F-8342 (Czech Collection of Microorganisms) were observed by macrodilution method on solidified potato-dextrose growth medium (PDA). During culturing, the diameters of growing fungal colonies were measured at regular intervals [27, 28]. Briefly, the antimicrobial activity (bacteria and yeasts) gallium(III) complexes/ ligands were added to the microbial culture at the beginning of the cultivation-to the lag phase. Pure gallium(III) complexes/ligands were dissolved in DMSO. The final concentration of DMSO never exceeded 1.0 vol. % neither in control nor treated samples. Concentration of gallium(III) complexes used in experimental work for evaluation of antimicrobial activity was used in the range of 0.05–2 mM, for the free ligands in the range of 0.5–2.0 mM in all experiments. The antimicrobial activity of tested compounds was characterized by IC_50_ values (concentration of a compound that inhibits the growth of model microorganism on 50% when compared to the untreated control). The IC_50_ values were evaluated from toxicity curves. All the obtained results of antimicrobial activity were compared to the activity of Ga(NO_3_)_3_.

### Anticancer activity

#### Cytotoxic activity of Ga(III) complexes

Cell culture the cell lines A2058 (human metastatic melanoma), BLM (human metastatic melanoma), HCT116 (human colorectal carcinoma), HeLa (human cervical adenocarcinoma), Jurkat (human leukaemic T cell lymphoma). PaTu 8902 (human pancreatic adenocarcinoma), MDA-MB-231 (human mammary gland adenocarcinoma) and human dermal fibroblasts (HDF) were obtained from (ATCC, Manassas, VA, USA). HeLa, HCT, Jurkat and MDA-MB-231 cells were cultured in growth medium RPMI 1640 (Biosera, Kansas City, MO, USA) supplemented with a 10% foetal bovine serum (FBS) (Invitrogen, Carlsbad, CA, USA) and 1X HyClone™ Antibiotic/Antimycotic Solution (GE Healthcare, Piscataway, NJ, USA). BLM, A2058, PaTu 8902 and HDF cells were cultured in growth medium consisting of high glucose Dulbecco´s Modified Eagle Medium (DMEM) + sodium pyruvate (Biosera) supplemented with a 10% FBS, Antibiotic/Antimycotic solution and 25 mM HEPES (only PaTu 8902). Cells were maintained in standard conditions with an atmosphere containing 5% CO_2_ at 37 °C. Prior to each experiment, cell viability was greater than 95%.

### MTS assay

The antiproliferative activity of Ga(III) complexes GaPic, GaDpic, GaChel, GaCldpic, free ligands (HPic, H_2_Dpic, H_2_Chel, H_2_Cldpic) and cisplatin (cisPt) were evaluated by colorimetric microculture MTS (3-(4,5-dimethylthiazol-2-yl)-5-(3-carboxymethoxyphenyl)-2-(4-sulfophenyl)-2H tetrazolium) assay (Promega, Madison, WI, USA). Cancer and non-cancer cells were seeded at density 5 × 10^3^ cells/well in 96-well polystyrene microplates. Twenty-four hours after cell seeding, different concentrations (1.0—100.0 µM) of the compounds were tested. After 72 h of incubation, 10 µL of MTS was added to each well. After an additional 2 h, cell proliferation was evaluated by measuring of absorbance at wavelength 490 nm using the automated Cytation™ 3 Cell Imaging Multi-Mode Reader (Biotek, Winooski, VT, USA). The absorbance of control wells was taken as 100%, and the results were expressed as a percent of untreated control. IC_50_ values were calculated from MTS analyses.

### Cytotoxic effect of GaPic and its Ag(I) and Zn(II) analogues against HepG2 cell line

#### Cell culture

HepG2 cells were cultivated in MEM medium supplemented with 10% foetal bovine serum (FBS) and 1% penicillin–streptomycin, 1 μg/mL gentamycin and 1 mM sodium pyruvate at 37 °C in a humidified atmosphere with 5% CO_2_.

Cell passage was performed in a small Petri dish every third day. After removal of old medium cells were washed twice with 2 ml of sterile, serum-free medium, then 2 ml of pre-heated trypsin containing medium were added and cells were placed in the CO_2_ incubator. After 10 min of incubation, we observed the detachment of the adherent cells with the naked eye and 3 ml of MEM medium were added to them. The passage was performed aseptically in a laminar box with a vertical air flow.

#### Cell viability assessment

Prior to each experiment, cell viability was determined using an automated cell counter (Countess II FL, Thermo Fisher Scientific, USA). 50 μl of the HepG2 cell suspension was transferred into new tube and 12.5 μl of trypan blue was added. After one minute, 10 μl was taken from the prepared suspension to determine the number of viable cells in 1 ml. The automated cell counter determined the total number of viable cells and the percentage of viability.

#### MTT test

3-(4,5-dimethylthiazol-2-yl)-2,5-diphenyl tetrazolium bromide (MTT) assay was performed to assess the effect of metal complexes on cell viability. Five different concentrations (10, 25, 50, 75, 100 µM) of AgPic, ZnPic, GaPic complexes, picolinic acid (HPic) and gallium(III) nitrate as a standard were used for HepG2 cells. Cells (2 × 10^4^/200 μl) were incubated in 96-well microtiter plates for 24 and 48 h in the presence of metal complexes, picolinic acid, and gallium(III) nitrate, at 37 °C in humified atmosphere with 5% CO_2_. The cells were centrifuged for 10 min at 2500 rpm and 20 °C, supernatant was removed and 180 μl of fresh serum-free medium was added. Then 10 μl of MTT solution with a concentration of 5 mg/ml was added and cells were incubated for next 2–3 h in the dark until a precipitate formed. After centrifugation and removal of supernatant 150 μl of DMSO was added to the cells to dissolve the precipitate. Absorbance at 540 nm was measured using a spectrophotometer.

### Fluorescence spectroscopy

Fluorescence measurements were carried out on a Varian Cary Eclipse spectrofluorometer. The quenching studies of BSA with the Ga(III) complexes were investigated to determine whether they were able to quench fluorescence of BSA. All measurements were performed at 25 °C in 0.01 M PBS buffer (pH 7.4). The fluorescence spectra were measured at an excitation wavelength of 280 nm, with a slit width of 10 nm for the excitation and emission beams, in the range of 300–450 nm and analysed according to the classical Stern–Volmer Equation:

$$F_{0} / \, F = { 1} + K_{{{\text{SV}}}} \left[ Q \right]$$

where *F*_*0*_ and *F* are the fluorescence intensities in the absence and the presence of the quencher, respectively, *K*_SV_ is the Stern–Volmer constant, and [*Q*] is the concentration of quencher [9].

## Results and discussion

### Syntheses

**Scheme 1:**
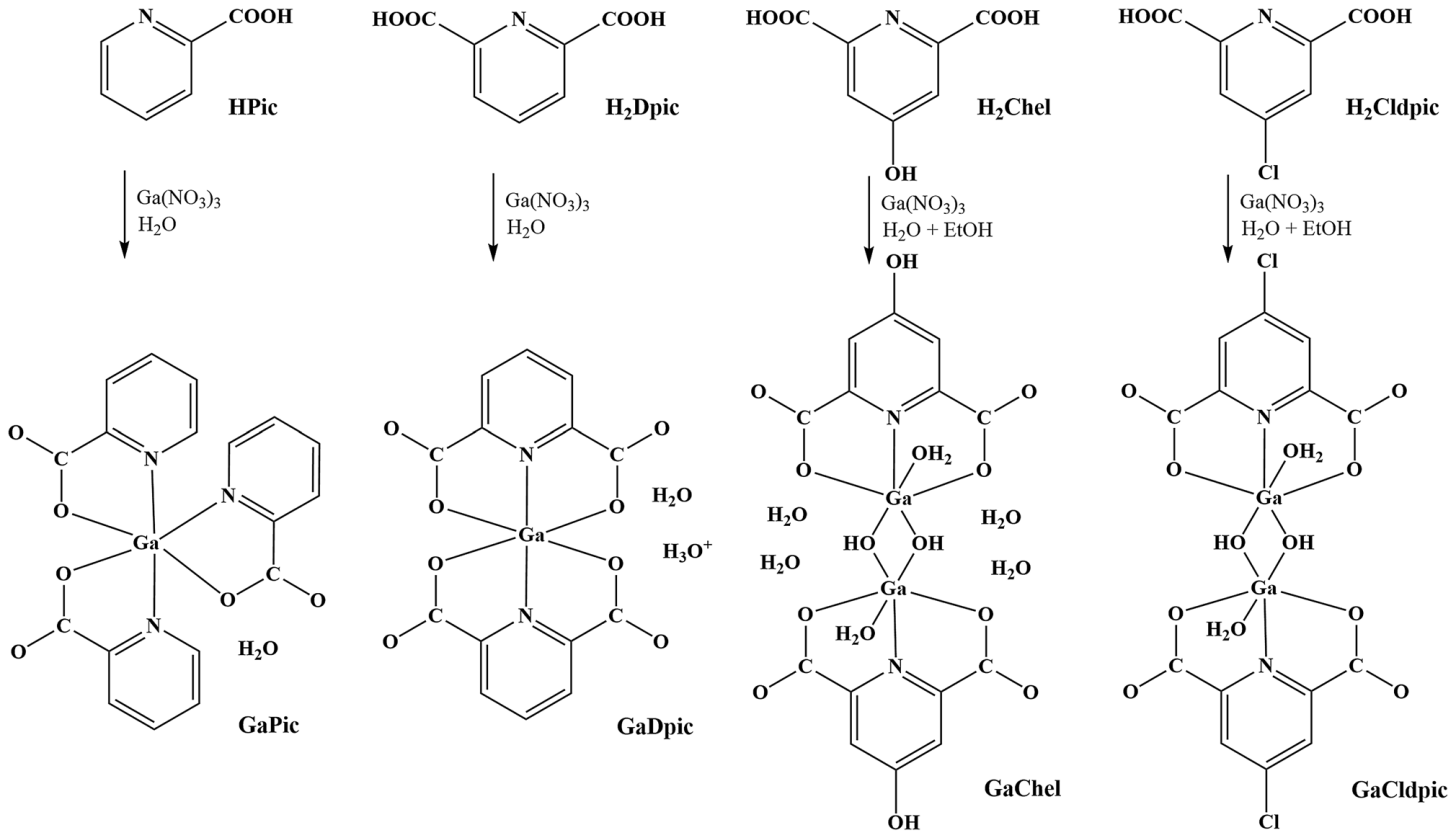
Synthetic procedure used for the synthesis of gallium(III) complexes

Compounds GaPic and GaDpic were synthesized from aqueous solution of Ga(NO_3_)_3_ and HPic/H_2_Dpic at a 1:2 molar ratio of Ga(NO_3_)_3_: HPic/H_2_Dpic. After slow evaporation (two weeks) at room temperature, small colourless crystals of compounds were formed. In the case of compounds GaChel and GaCldpic**,** ethanol: water mixture (1:3) was used to dissolve acids H_2_Chel and H_2_Cldpic and 1:1 molar ratio ((GaNO_3_)_3_: acid) was used to obtain crystalline products without ligand crystallisation in the solution. After 10 days of slow evaporation, light brown crystals of GaChel and yellowish crystals of GaCldpic were formed (Scheme 1). We conducted other experiments with the different Ga(NO_3_)_3_: acid molar ratios (1:3 for GaPic; 1:1 for GaDpic; 1:2 for GaChel and GaCldpic) used. However, structural motifs of solid products are identical in all the attempts. In addition, the raise of pH of the solution H_2_Dpic to 4 yielded the same product GaDpic, but with worst quality of crystals. All the complexes are air-stable, soluble in DMSO, GaCldpic is slightly soluble in ethanol.

## Solid state study

### IR spectra

The IR spectra of GaPic, GaDpic, GaChel, GaCldpic complexes and the free acids HPic, H_2_Dpic, H_2_Chel and H_2_Cldpic are shown in Fig. S1 and characteristic absorption bands are given in Table S2. Infrared spectra of pyridine carboxylic acids and of their metal complexes are relatively well discussed in the literature [29–31] and theoretical and experimental correlations of the acids spectra are also discussed [32, 33]. In the spectra of the complexes, absorption bands of the O–H stretching vibrations in the range of 3615—3225 cm^−1^ and H_2_O deformation vibrations in the range of 1639–1587 cm^−1^ are visible (Table S2), confirming the presence of water molecules in the structure. Absorption bands corresponding to the asymmetric and symmetric stretching vibrations of carboxylate group are present in the spectra of the complexes due to the carboxylic group deprotonation [34]. Based on the wavenumber difference Δ(*ν*_as_–*ν*_s_), it is possible to predict the coordination mode of the carboxylate group [35]. ∆ values are relatively high for the prepared gallium(III) complexes (Δ > 260 cm^−1^, Table S2), predicting the *O*-monodentate mode of carboxylate group coordination [35], in accordance with the X-ray analysis data. The ring breathing vibration usually observed about 1000 cm^−1^ in the IR spectra of pyridinedicarboxylic acids [29, 33] is shifted by 20–50 cm^−1^ in gallium(III) complexes spectra (Table S2). Similarly the mentioned vibration shift was observed in other metal pyridine carboxylates, e. g. [Mn(Pic)_2_]_*n*_ [36], [Zn(Pic)_2_(H_2_O)_2_]⋅2H_2_O [37] or [Cu(Chel)(H_2_O)(pym)]·H_2_O (pym = 2-pyridylmethanol) [38], where also the pyridine ring nitrogen atom is metal coordinated. Moreover, characteristic in-plane *β*(CCH) vibrations in the range of 1300–1060 cm^−1^ and out-of-plane *γ*(CCH) vibrations in the range of 780–640 cm^−1^ are also identified in all the spectra (Table S2).

## Thermal analysis

To estimate the thermal stability of the complexes thermogravimetric analysis was carried out under the air atmosphere and obtained TG curves of GaPic and GaDpic are shown in Fig. S2 and of GaChel and GaCldpic in Fig. S3. The first step of thermal degradation of GaPic and GaDpic complexes is the dehydration process, which occurs above 50 °C and is accompanied by the release of one molecule of crystal water in GaPic with mass loss of 4.01% (clcd. mass loss 3.97%), in the case of GaDpic H_2_O and H_3_O^+^ molecules are released with mass loss of 8.59% (clcd. mass loss 8.48%). The anhydrous complex [Ga(Pic)_3_] is stable in the temperature range of 130—345 °C (Fig. S2). Above 345 °C, the organic part is released (exp. mass loss 74.25%, clcd. 75.39%) in two steps. The weight loss in the first step in the range of 345—430 °C is more intense due to the release of two Pic^−^ ligands and the final mass loss of the organic part occurs in a wider temperature range of 430—610 °C. The thermal decomposition of the anhydrous intermediate [Ga(Dpic)_2_]^−^ in the temperature range of 140—750 °C corresponds to the multi-step decomposition of the organic part with mass loss of 68.18% (clcd. 68.22%). The final decomposition product of both the complexes is Ga_2_O_3_ with residual mass of 21.74% for GaPic (clcd. residual mass 20.64%) and of 23.19% for GaDpic (residual mass of 21.45%).

Comparing the thermal stability of anhydrous silver(I) [6], zinc(II) [39] and the gallium(III) picolinates, it can be concluded that the prepared gallium(III) complexes are more stable than silver(I) and zinc(II) analogues. Moreover, dehydrated metal picolinate complexes are generally more thermally stable than metal dipicolinate complexes.

Similarly, dehydration process is the first step of the thermal decomposition of GaChel complex (Fig. S3), it takes place in the range of 55–209 °C and corresponds to the release of 4 molecules of crystal water with mass loss of 11.34% (clcd. 11.19%). In the next step, up to 299 °C, two aqua and two hydroxide ligands are released (exp. mass loss 9.28%, clcd. 10.88%). The release of the organic part occurs in the temperature range of 300–576 °C and the final decomposition product is Ga_2_O_3_ with residual mass of 32.41% (clcd. 29.12%). GaCldpic does not contain crystal water in the structure and is thermally stable up to 221 °C, above this temperature two aqua and two hydroxide ligands are released (exp. mass loss 9.68%, clcd. 11.51%) up to 315 °C (Fig. S3). In the temperature range of 315–640 °C, the organic part is released and the final residue represents Ga_2_O_3_ (residual mass 34.28%, clcd. 30.80%). Even when comparing the thermal stability of the dehydrated form of the silver(I) complex AgCldpic (AgCldpic = [Ag(H_2_Cldpic)(HCldpic)]·2H_2_O) (155 °C) [18] and of the dehydrated form of the prepared GaCldpic (221 °C), the trend is similar as was observed in the case of metal picolinates.

## Crystal structure of complex GaPic

**Fig. 1:**
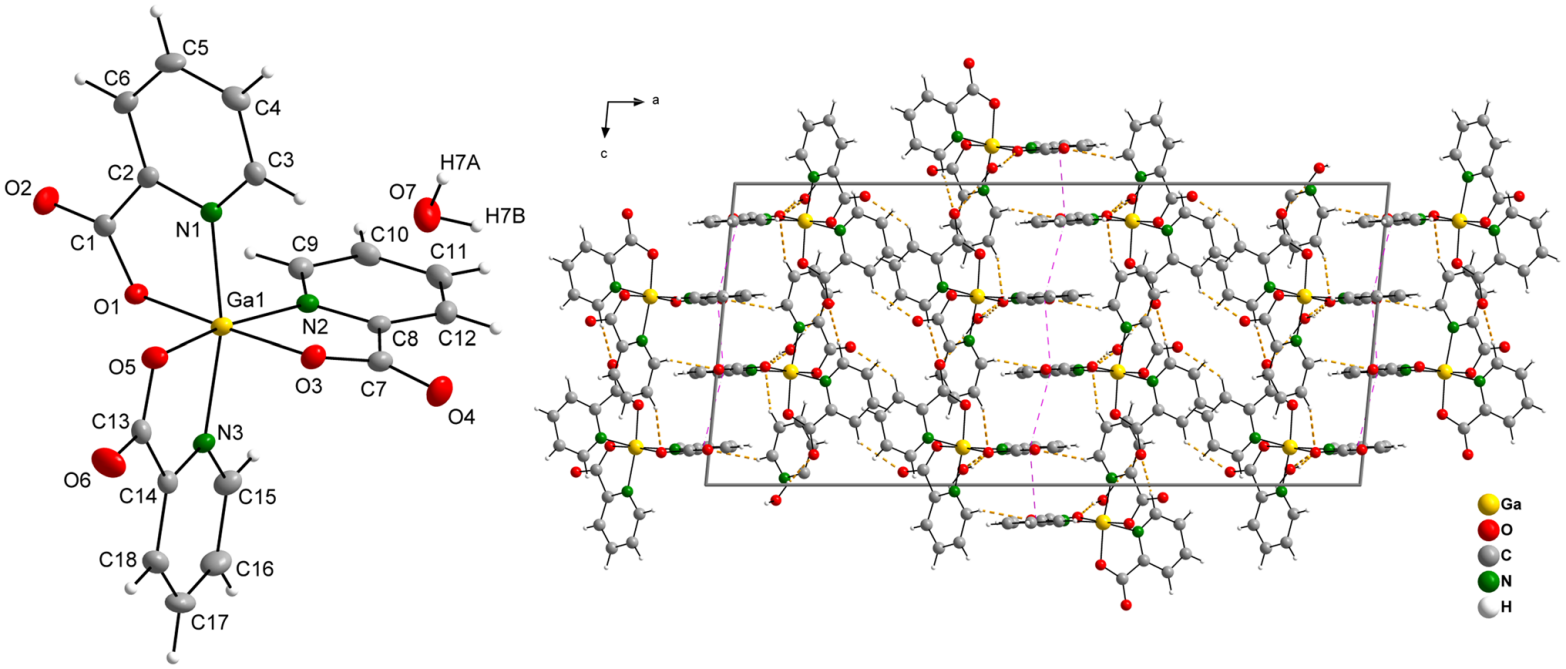
Molecular structure and atomic labelling scheme for complex [Ga(Pic)_3_]·H_2_O (GaPic) with ellipsoids drawn at the 50% probability level (left) and crystal packing diagram viewed along [0 1 0] with hydrogen bond system (orange dashed lines) and π-π stacking interactions (purple dashed lines) (right)

Complex GaPic crystallizes in monoclinic lattice with space group *C*2/*c*. The metal ion is bound to three *N,O-*bidentate chelating Pic^−^ ligands showing distorted octahedral geometry (see Table 1) with meridional stereochemistry. The bite angle is ~ 81°, which has been observed also in Rh(III) or Ir(III) picolinato complexes with analogous molecular structure [40]. On the other hand, in organometallic compound [(CH_3_)_2_ Ga(Pic)]_2_ [41], the involvement of the carboxylate oxygen atom to two different coordination modes (*N,O*-chelation and oxo-bridging coordination) resulted in the bite angle decrease (less than 78°) and an increase of the Ga–N bond lengths (2.178(4); 2.149(4) Å) compared to the parameters in GaPic (Table 1). There is a single molecule of crystal water present (Fig. 1), which is hydrogen-bonded to the carboxylate oxygen atoms of two individual complex molecules [Ga(Pic)_3_], as was observed also in other metal picolinates [M(Pic)_3_]·H_2_O (M = Rh(III); Ir(III)) [40]. Geometrical parameters characterizing hydrogen bonds (Fig. 1, orange dashed lines) are summarized in Table S3. In addition, π-π stacking interactions are observed in the structure (Fig. 1, purple dashed lines), with commonly observed values for aromatic nitrogen heterocycles [42] and their geometrical parameters are listed in Table S4.

**Table 1:** Selected bond distances and angles (Å, °) for GaPic

Ga1–O3	1.9478(12)	O3–Ga1–O5	89.35(5)
Ga1–O5	1.9557(12)	O3–Ga1–O1	174.62(5)
Ga1–O1	1.9602(12)	O5–Ga1–O1	96.02(5)
Ga1–N1	2.0480(14)	O3–Ga1–N1	99.39(5)
Ga1–N2	2.0726(14)	O5–Ga1–N1	88.86(5)
Ga1–N3	2.0761(14)	O1–Ga1–N1	81.11(5)
O1–C1	1.306(2)	O3–Ga1–N2	81.11(5)
C1–O2	1.214(2)	O5–Ga1–N2	170.24(5)
O3–C7	1.300(2)	O1–Ga1–N2	93.51(5)
C7–O4	1.210(2)	N1–Ga1–N2	94.73(5)
O5–C13	1.289(2)	O3–Ga1–N3	90.05(5)
C13–O6	1.219(2)	O5–Ga1–N3	81.55(5)
		O1–Ga1–N3	90.40(5)
		N1–Ga1–N3	166.49(6)
		N2–Ga1–N3	96.28(5)

### Crystal structure of complex GaDpic

**Fig. 2:**
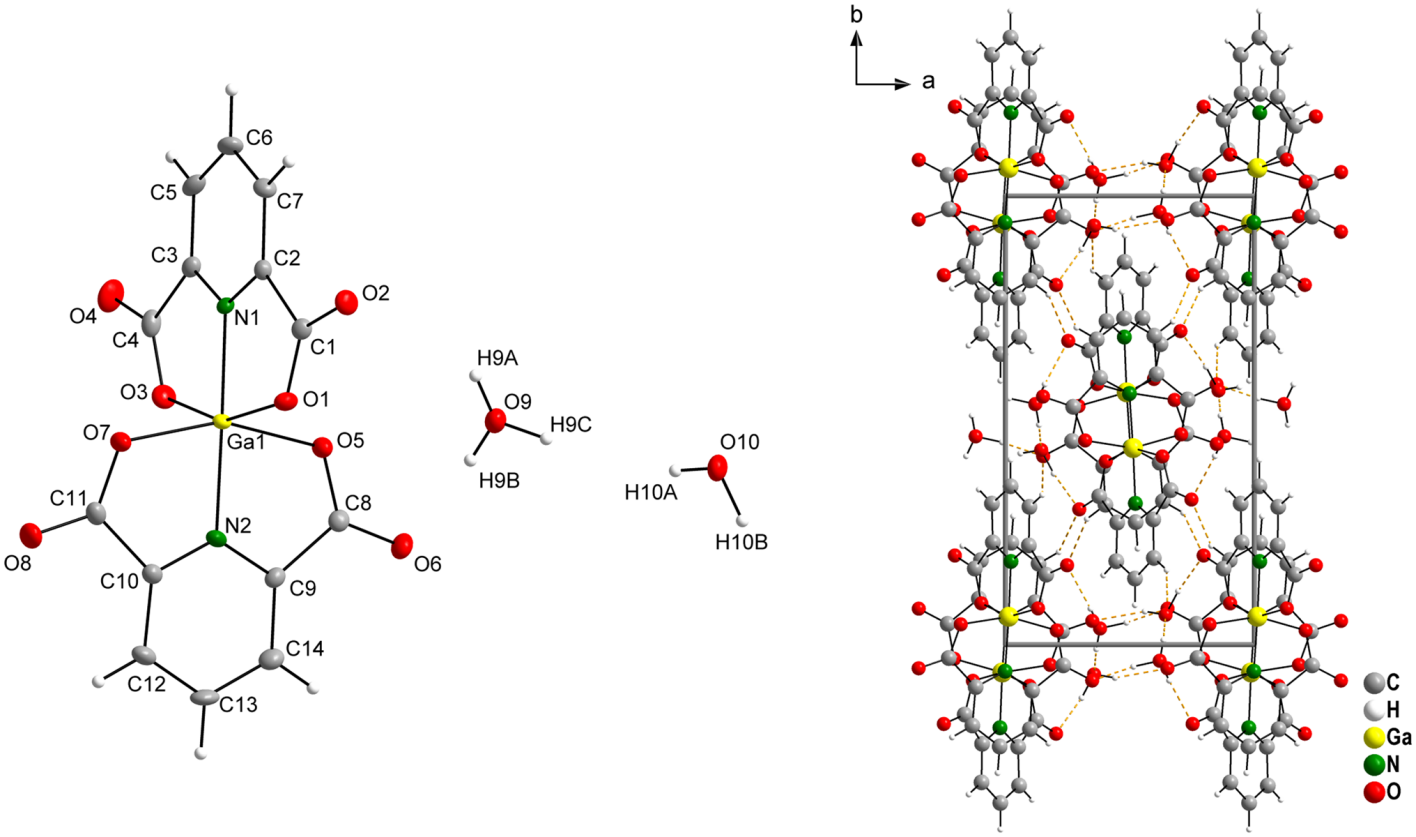
Molecular structure and atomic labelling scheme for complex H_3_O[Ga(Dpic)_2_]·H_2_O (GaDpic) with ellipsoids drawn at the 50% probability level (left) and crystal packing diagram viewed along [0 0 1] with hydrogen bond system (orange dashed lines) (right)

Complex GaDpic crystallizes in orthorhombic lattice with space group *Pna*2. Gallium(III) is hexacoordinated by two dianionic Dpic^2−^ ligands, which act as tridentate chelating ligands forming distorted octahedral geometry. There is one crystal water molecule and one oxonium counterion in the structure (Fig. 2). The Ga–O and Ga–N bond lengths as well the bond lengths of Dpic^2−^ are in agreement with the published values for other gallium(III) pyridinedicarboxylates [43–46]. The deviation from the ideal linearity of the angle N1–Ga1–N2 is only 1.57°, which is a relatively small deviation compared to the deviations from the linearity of the mentioned angle in (pipzH_2_)_0.5_[Ga(Dpic)_2_]·H_2_Dpic·2H_2_O (pipz = piperazine) with the deviation of 8.89° [44], in (dmpH)[Ga(Dpic)_2_]⋅2H_2_O (dmp = 2,9-dimethyl-1,10-phenanthroline) with the deviation of 8.73° [45] or in (pydaH)[Ga(Dpic)_2_]·3,25H_2_O·CH_3_OH (pyda = 2,6-pyridinediamine) with the deviation of 12.88° [43]. Selected bond lengths and angles are given in Table 2.

**Table 2:** Selected bond distances and angles (Å, °) for GaDpic

Ga1–N1	1.9576(15)	N1–Ga1–N2	178.43(12)
Ga1–N2	1.9819(17)	N1–Ga1–O5	101.42(16)
Ga1–O5	2.0027(18)	N2–Ga1–O5	78.83(11)
Ga1–O7	2.0028(18)	N1–Ga1–O7	101.09(16)
Ga1–O3	2.0102(19)	N2–Ga1–O7	78.69(11)
Ga1–O1	2.0355(17)	O5–Ga1–O7	157.48(7)
		N1–Ga1–O3	79.43(11)
		N2–Ga1–O3	99.01(9)
		O5–Ga1–O3	92.84(8)
		O7–Ga1–O3	91.99(8)
		N1–Ga1–O1	78.93(11)
		N2–Ga1–O1	102.64(8)
		O5–Ga1–O1	89.33(7)
		O7–Ga1–O1	94.25(8)
		O3–Ga1–O1	158.25(7)

The counter ion H_3_O^+^ and water molecule play an important role in the 3D network formation. The O–H···O and weak C–H···O hydrogen interactions are shown in Fig. 2 as orange dashed lines and their geometric parameters are given in Table S5.

### Crystal structure of complex GaChel

**Fig. 3:**
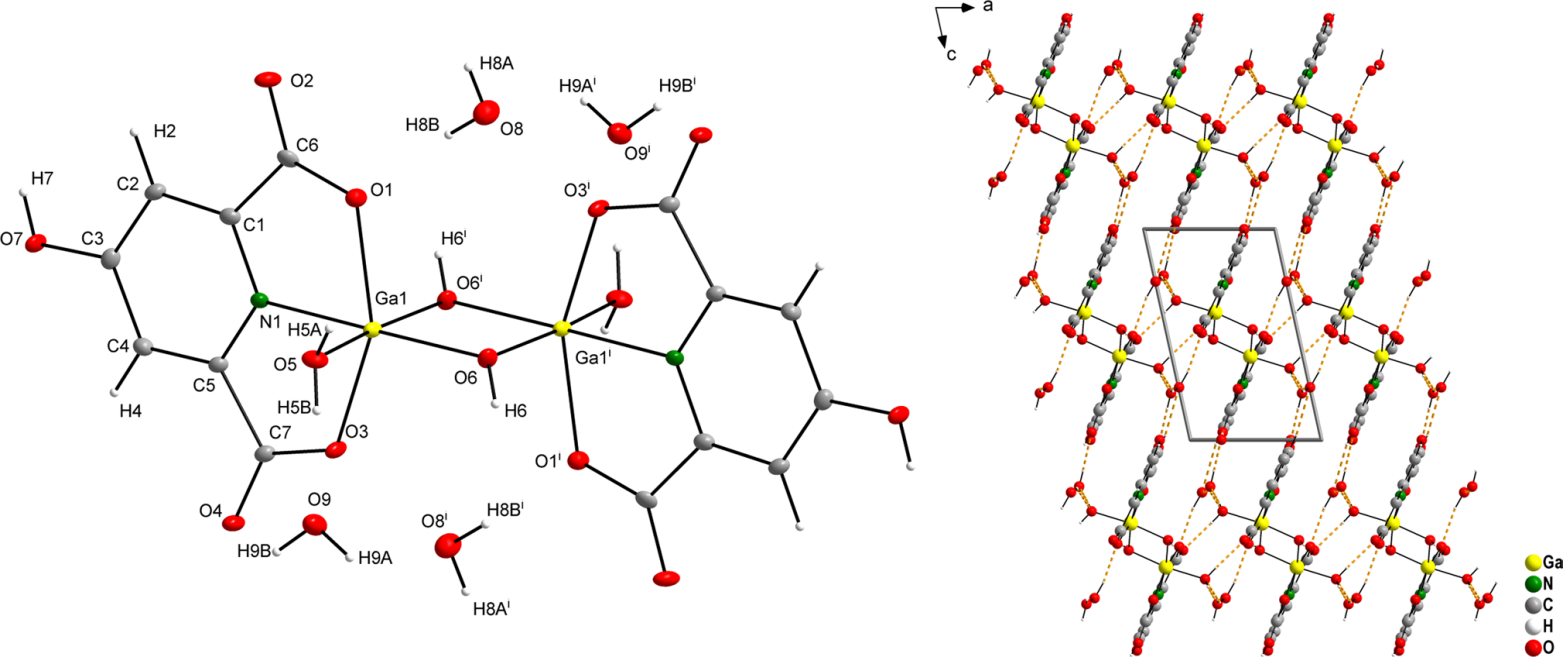
Molecular structure and atomic labelling scheme for complex [Ga(Chel)(H_2_O)(OH)]_2_·4H_2_O (GaChel) with ellipsoids drawn at the 50% probability level (left) and crystal packing diagram viewed along [0 1 0] with hydrogen bond system (orange dashed lines) (right) (*i* = −*x* + 1, −*y*, -*z* + 1)

Complex GaChel crystallizes in triclinic lattice with space group *P*. The structure of GaChel consists of centrosymmetric [Ga(Chel)(H_2_O)(OH)]_2_ dimeric units, where two crystallographically equivalent Ga(III) ions are bridged by two hydroxyl groups. Four molecules of crystal water are also present in the structure. The Chel^2−^ ligand acts as *O,N,O*-tridentate chelating ligand and the remaining three coordination sites are occupied by two oxygen atoms from two hydroxide-bridging ligands and by one atom from aqua ligand (Fig. 3), resulting in distorted octahedral geometry. The same structural motif was observed in Fe(III) complex [Fe(Chel)(H_2_O)(OH)]_2_·4H_2_O [47] and Cr(III) complex [Cr(Chel)(H_2_O)(OH)]_2_·4H_2_O [48]. Selected bond lengths and bond angles for GaChel are given in Table 3. The largest deviation from ideal octahedral symmetry was observed for the angle O1–Ga1–O3 (∢ = 156.20(6) Å), similarly as was observed in the above-mentioned isostructural Fe(III) [47] and Cr(III) compounds [48] as well in the prepared GaCldpic complex and the observed deviation is attributed to the limited bite of the tridentate ligand [48].

**Table 3:** Selected bond distances and angles (Å, °) for GaChel

Ga1–O6	1.8897(15)	O6–Ga1–N1	172.15(7)
Ga1–N1	1.9733(17)	O6–Ga1–O6^i^	78.76(7)
Ga1–O6^i^	1.9827(15)	N1–Ga1–O6^i^	93.71(7)
Ga1–O5	1.9965(16)	O6–Ga1–O5	92.82(7)
Ga1–O3	2.0027(15)	N1–Ga1–O5	94.80(7)
Ga1–O1	2.0526(15)	O6 ^i^–Ga1–O5	171.24(6)
		O6–Ga1–O3	99.51(6)
		N1–Ga1–O3	78.74(6)
		O6 ^i^–Ga1–O3	94.60(6)
		O5–Ga1–O3	89.11(7)
		O6–Ga1–O1	104.15(6)
		N1–Ga1–O1	78.16(6)
		O6 ^i^–Ga1–O1	92.52(6)
		O5–Ga1–O1	87.19(7)
		O1–Ga1–O3	156.20(6)

Symmetry transformations used to generate equivalent atoms: (i) −*x* + 1, −*y*, −*z* + 1

The extensive hydrogen bond network (Fig. 3., orange dashed lines) is formed by O–H···O and C–H···O interactions involving the coordinated and free water molecules, hydroxide-bridging ligands as well as hydroxy and carboxylate groups of Chel^2−^ ligand. Geometric parameters of hydrogen interactions are given in Table S6.

### Crystal structure of complex GaCldpic

**Fig. 4:**
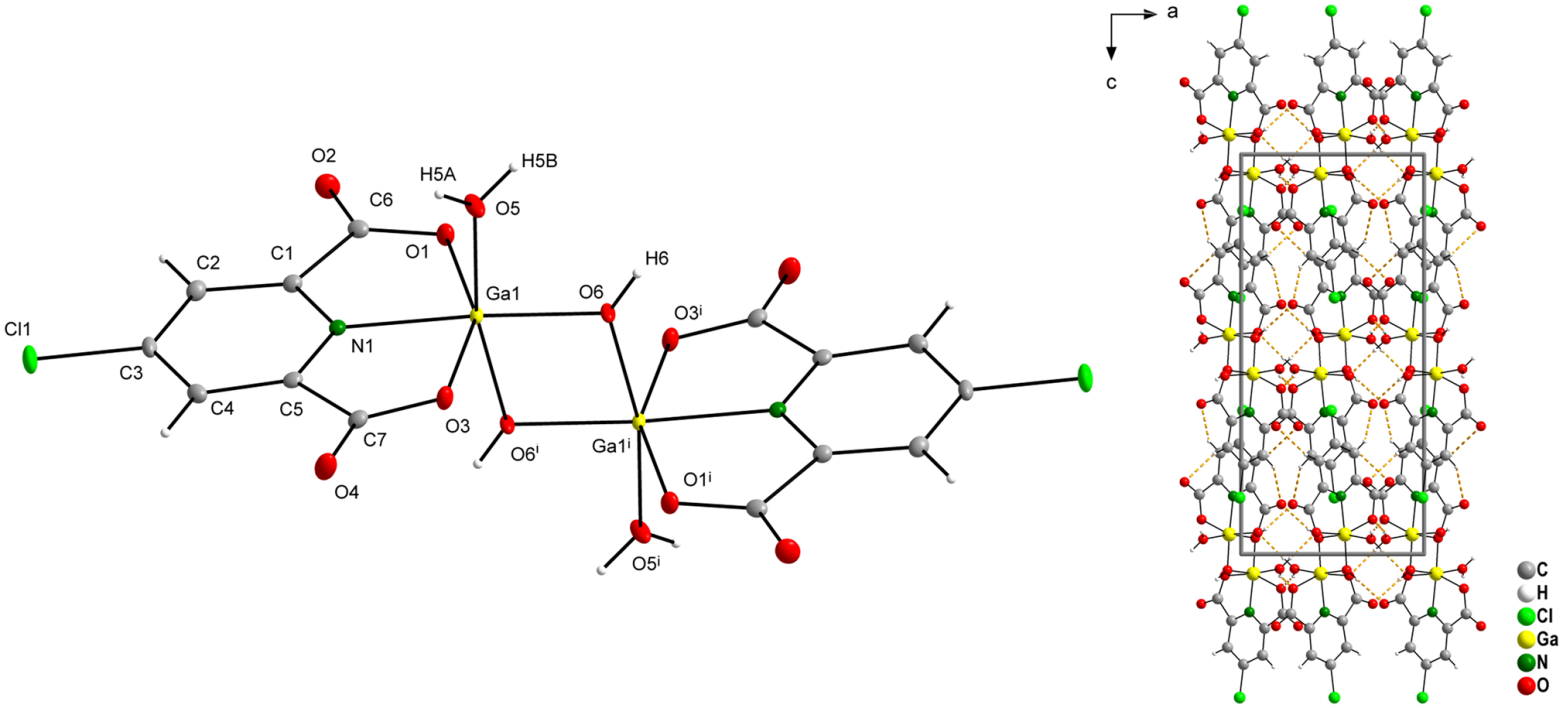
Molecular structure and atomic labelling scheme for complex [Ga(Cldpic)(H_2_O)(OH)]_2_ (GaCldpic) with ellipsoids drawn at the 50% probability level (left) and crystal packing diagram viewed along [0 1 0] with hydrogen bond system (orange dashed lines) (right) (*i* = −*x*, −*y* + 1, −*z*)

Complex GaCldpic crystallizes in orthorhombic lattice with space group *Pbca*. The structure consists, similarly to the GaChel structure, of centrosymmetric [Ga(Cldpic)(H_2_O)(OH)]_2_ dimeric units (Fig. 4). The isostructural motif of dimeric units was observed in Cr(III) compound [Cr(Cldpic)(H_2_O)(OH)]_2_·2H_2_O [48]. Selected bond lengths and angles are given in Table 4. The angle O1–Ga1–O3 with the value 155.02(4)° also has the greatest contribution to the deformation of the octahedral coordination polyhedron, as was also observed in [Cr(Cldpic)(H_2_O)(OH)]_2_·2H_2_O (∢ = 156.7(1)°) [48]. The geometrical parameters of the structure stabilising O–H···O and C–H···O hydrogen interactions shown by orange dashed lines in Fig. 4 are given in Table S7.

**Table 4:** Selected bond distances and angles (Å, °) for GaCldpic

Ga1–O6	1.8791(11)	O6–Ga1–O6^i^	76.81(5)
Ga1–N1	1.9932(12)	O6–Ga1–O5	90.88(5)
Ga1–O6^i^	1.9411(11)	O6^i^–Ga1–O5	167.46(5)
Ga1–O5	1.9610(11)	O6–Ga1–N1	174.13(5)
Ga1–O3	2.0858(11)	O6^i^–Ga1–N1	97.55(5)
Ga1–O1	2.0425(10)	O5–Ga1–N1	94.81(5)
		O6–Ga1–O1	100.31(5)
		O6^i^–Ga1–O1	93.46(5)
		O5–Ga1–O1	91.12(5)
		N1–Ga1–O1	78.19(5)
		O6–Ga1–O3	104.67(5)
		O6^i^–Ga1–O3	92.53(5)
		O5–Ga1–O3	88.18(5)
		N1–Ga1–O3	76.99(5)
		O1–Ga1–O3	155.02(4)

Symmetry transformations used to generate equivalent atoms: (i) −*x*, −*y* + 1, −*z*

In GaCldpic as well in GaChel structures, inequivalent Ga–O(H) bond lengths are observed due to the presence of different groups (pyridine nitrogen atom and water molecule) located in *trans* positions relative to M–O(H) bonds [47].

### Complexes stability in solution

The stability of the prepared complexes in 1% DMSO-*d*_6_/D_2_O solution was tested using ^1^H NMR spectroscopy to evaluate their suitability for in vitro biological testing. The ^1^H time-dependent spectra of complexes are shown in Fig. S4A–D. The assignment of chemical shift values ​​to the signals is given in the figure captions in the ESI and for comparison, ^1^H NMR spectra of appropriate free ligands with the pH adjusted to the same value as the solutions of the complexes are also included in the figures. From the ^1^H time-dependent spectra of GaPic (Fig. S4A) it is clear that there are inequivalent protons of the picolinate ligands present in the solution as Thottathil et al. also found out [49]. As mentioned above, the broadened non-splitting signals suggest that changes occur over time due to the fluxionality of the ligand in the solution. Moreover, by comparing GaPic spectra with the spectrum of pure HPic acid, it is evident that GaPic still remains in the form of a complex species in solution and does not decompose over the period of 96 h.

Comparing GaDpic, GaChel and GaCldpic complexes behaviour in the observed period (96 h), the changes in chemical shifts and signal intensity in time-dependent NMR spectra are not observed (Fig. S4B-D). Therefore the prepared compounds were considered to be stable in 1%-DMSO-d_6_/D_2_O solution for 96 h.

## Solution measurements

A dissociation constant for picolinic acid with a value of p*K*_*1*_ = 1.17 corresponds to the deprotonation of the carboxyl group and dissociation constant p*K*_*2*_ = 5.25 to the deprotonation of the nitrogen atom of the pyridyl ring. Both dissociation constants correspond with previously determined data [50, 51]. Comparing this value with dissociation constant of the nicotinic (p*K*_*2*_ = 4.66) [50] and isonicotinic (p*K*_*2*_ = 4.89) [52] acids we found that value indicates the intensive involvement of a hydrogen atom in the intramolecular hydrogen bond of picolinic acid. Dipicolinic acid dissociation can be described as a two-step process with determined values ​​of dissociation constants p*K*_*2*_ = 2.02 and p*K*_*3*_ = 4.74. The value of p*K*_*2*_ corresponds to the deprotonation of the carboxylic group (H_2_Dpic ↔ HDpic^−^ + H^+^) and p*K*_*3*_ = 4.74 to the deprotonation of the pyridyl ring (HDpic^−^ ↔ Dpic^2−^ + H^+^). Under our experimental conditions, the first protonation constant could not be determined.

With the above-mentioned pyridine carboxylic acids, the stability constants for mononuclear complex species in the binary systems Ga(III)-HPic/H_2_Dpic by potentiometric data fitting using the calculation program OPIUM [23] were determined; [Ga(Pic)_2_]^+^ (log*β*_*021*_ = 16.23(6)) and [Ga(Pic)_3_] (log*β*_*031*_ = 20.86(2)), [Ga(Dpic)_2_]^−^ (log*β*_*021*_ = 15.42(9)) and [Ga(Dpic)_2_(OH)]^2−^ (log*β*_*-121*_ = 11.08(4)). Stability of our complexes is lower comparing to well-known GaM and KP46 complexes [53].

**Fig. 5:**
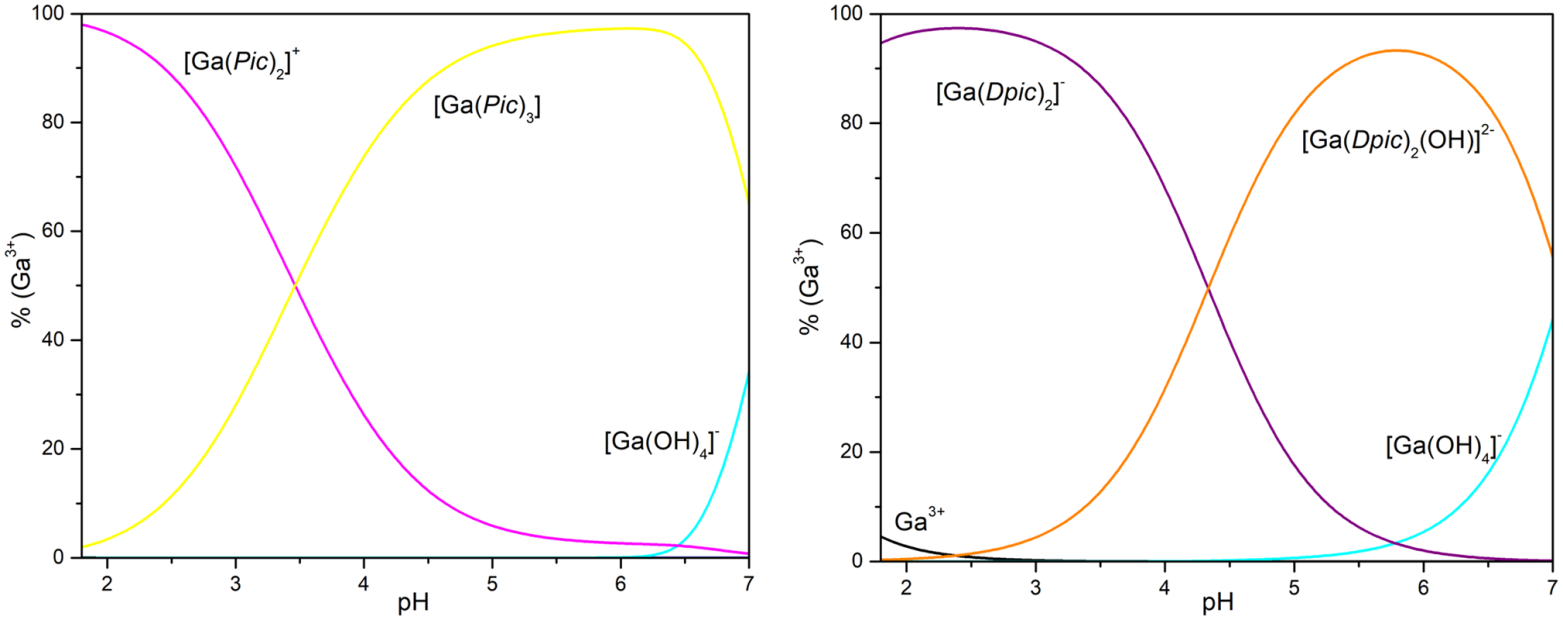
Distribution of Ga^3+^ species in the binary system Ga^3+^  + HPic in a molar ratio of 1: 4 (left, c_Ga_ = 1 mM, c_HPic_ = 4 mM) and Ga^3+^  + H_2_Dpic in a molar ratio of 1: 2 (right, c_Ga_ = 2 mM, c_H2DPic_ = 4 mM), *I* = 0.1 M, 25 °C

Figure 5 shows the complexing species distribution in dependence of pH values for the Ga(III)-HPic (molar ratio of 1: 4) and Ga(III)-H_2_Dpic systems (molar ratio of 1:2), respectively. It is clear that complex species [Ga(Pic)_2_]^+^ and [Ga(Dpic)_2_]^−^ occur immediately after the ligand addition to the acidified gallium(III) nitrate solution and their abundance is more than 90%. The [Ga(Pic)_3_] complex is present in the acidic solution in a low abundance and gradually its abundance increases with increasing pH value and becomes dominant from pH 3.5. Its high abundance, above 90%, can be observed in a wide pH range from 4.7 to 6.6. Complex species [Ga(Dpic)_2_(OH)]^2−^ inclusion to potentiometric data treatment provides the best fit for chemical model and its abundance is high (above 70%) already from pH 5.0. Similar behaviour was reported by Aghabozorg et al. [43].

Comparing stability constants of Ga-HPic binary systems with analogue systems for Al(III) (log*β*_*011*_ = 4.51, log*β*_*021*_ = 8.38, log*β*_*031*_ = 12.0; at 25 °C, *I* = 0.5 M) [51], we can conclude formation of more stable gallium(III) complex species. Moreover, Fe(III) forms less stable complex species with picolinate ligand (log*β*_*011*_ = 6.02 [54], log*β*_*021*_ = 12.88 [55], log*β*_*031*_ = 17.25 [56], but more stable complex for Fe(Dpic)_2_ complex species (log*β*_*021*_ = 17.13) [57] with dipicolinate ligand compared to Ga(III) analogues.

Two gallium(III) complex species Ga(Pic)_3_ and Ga(Dpic)_2_ have been isolated in crystalline form and their physicochemical and biological evaluation is described in the following paragraphs.

With the aim to confirm complexing species structures in solution ^1^H NMR spectra were measured by the procedure described in Experimental. Fig. S5 shows the ^1^H NMR spectra of picolinic acid (HPic) in the range of pH 2 to 11. The chemical shift changes depending on pH confirm the gradually picolinic acid dissociation and above pH 7 the completely deprotonated form of the Pic^−^ ligand is dominant in the solution. A comparison of potentiometric and ^1^H NMR data confirm their mutual agreement.

**Fig. 6:**
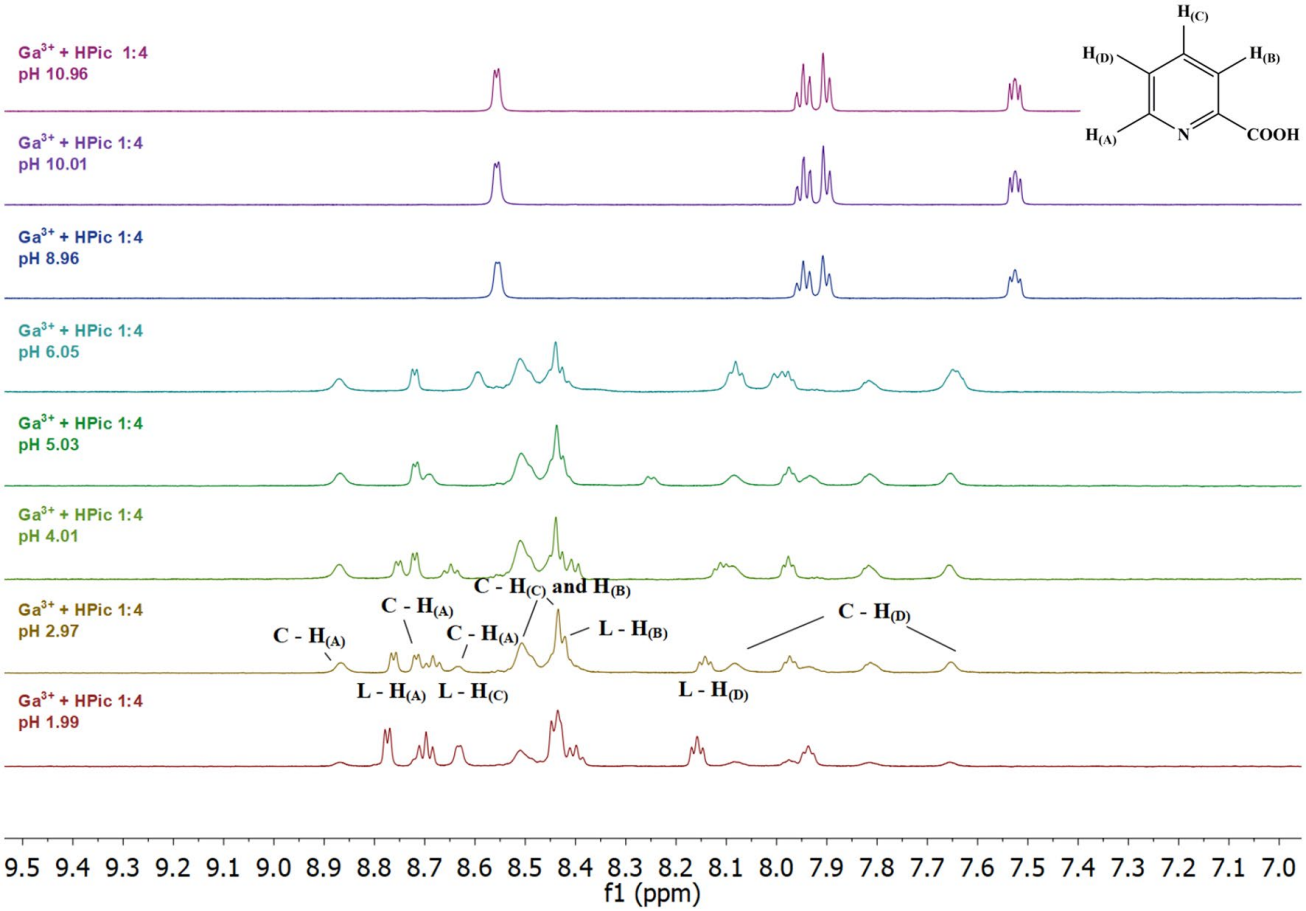
Dependence on pH of the ^1^H NMR spectra of an aqueous solution containing Ga(III) and HPic in a 1–4 ratio; c_Ga_ = 8 mM, c_HPic_ = 32 mM

After gallium(III) ion addition to the picolinic acid solution, we can observe significant changes in the pyridine hydrogen atom chemical shifts in the pH range from 2 to 6.0 (inclusive) (Fig. 6). Based on a detailed analysis of the spectra, these changes can be attributed to the formation of complex species, but at the same time it is possible to detect the presence of free acid in solution, which confirms the presence of signals in the spectra at pH 1.99 (δ_H_ 8.77 (d, *J* 5.7, H_A_), 8.69 (t, *J* 7.9, H_C_), 8.44 (d, *J* 7.9, H_B_), 8.15 (t, *J* 6.6, H_D_)), because their shape and position correspond to the signals in the spectrum of pure acid. Based on the results of potentiometric titrations of the binary system Ga(III)-HPic (distribution of complex species (Fig. 5)), these signals in the pH range from 2 to 6 can be assigned to [Ga(Pic)_2_]^+^ and [Ga(Pic)_3_] complex species in which protons are not equivalent and some signals may be the result of multiple peaks superposition, which may occur due to rotation of the complex species in solution. The protons of complexes [Ga(Pic)_2_]^+^ and [Ga(Pic)_3_] at ambient temperature give a very complex overlapped pattern, which is the result of the interaction of all species present in the solution. The present species affect not only the chemical shifts but also the transverse relaxation times T_2_ of the individual protons and thus the linewidth. Figure 6 shows that complexes [Ga(Pic)_2_]^+^ and [Ga(Pic)_3_] are not distinguishable. It seems likely that only minor structural differences in the arrangement of the ligands around the Ga(III) ions must occur. The spectra at pH 7 and 8 could not be interpreted because Ga(OH)_3_ precipitate gradually formed, which subsequently turned into a soluble [Ga(OH)_4_]^−^ species [58]. In the alkaline pH region, only the free acid signals (Pic^−^) were observed in the solution with the same chemical shifts as in the spectra of HPic. (δ_H_ 8.56 (d, *J* 4.8, H_A_), 7.94 (td, *J* 7.7, 1.7, H_C_), 7.90 (d, *J* 7.7, H_B_), 7.53 (t, *J* 6.6, H_D_)).

Fig. S6 shows the ^1^H NMR spectra of dipicolinic acid (H_2_Dpic) in the pH range from 2 to 11. Increasing pH causes deprotonation of the carboxylic group and the multiplet changes to two well-distinguished multiplets: at *δ*_H_ 8.60 (t, *J* 7.8, H_B_) and *δ*_H_ 8.42 (d, *J* 7.8, H_A_). As the pH increases, the chemical shift is changing to lower *δ*_H_ values (upfield, Fig. S6), and the spectra of the completely deprotonated form of dipicolinic acid are characterized by one multiplet signal centred at *δ*_H_ 8.00. Potentiometric and NMR data are in agreement.

**Fig. 7:**
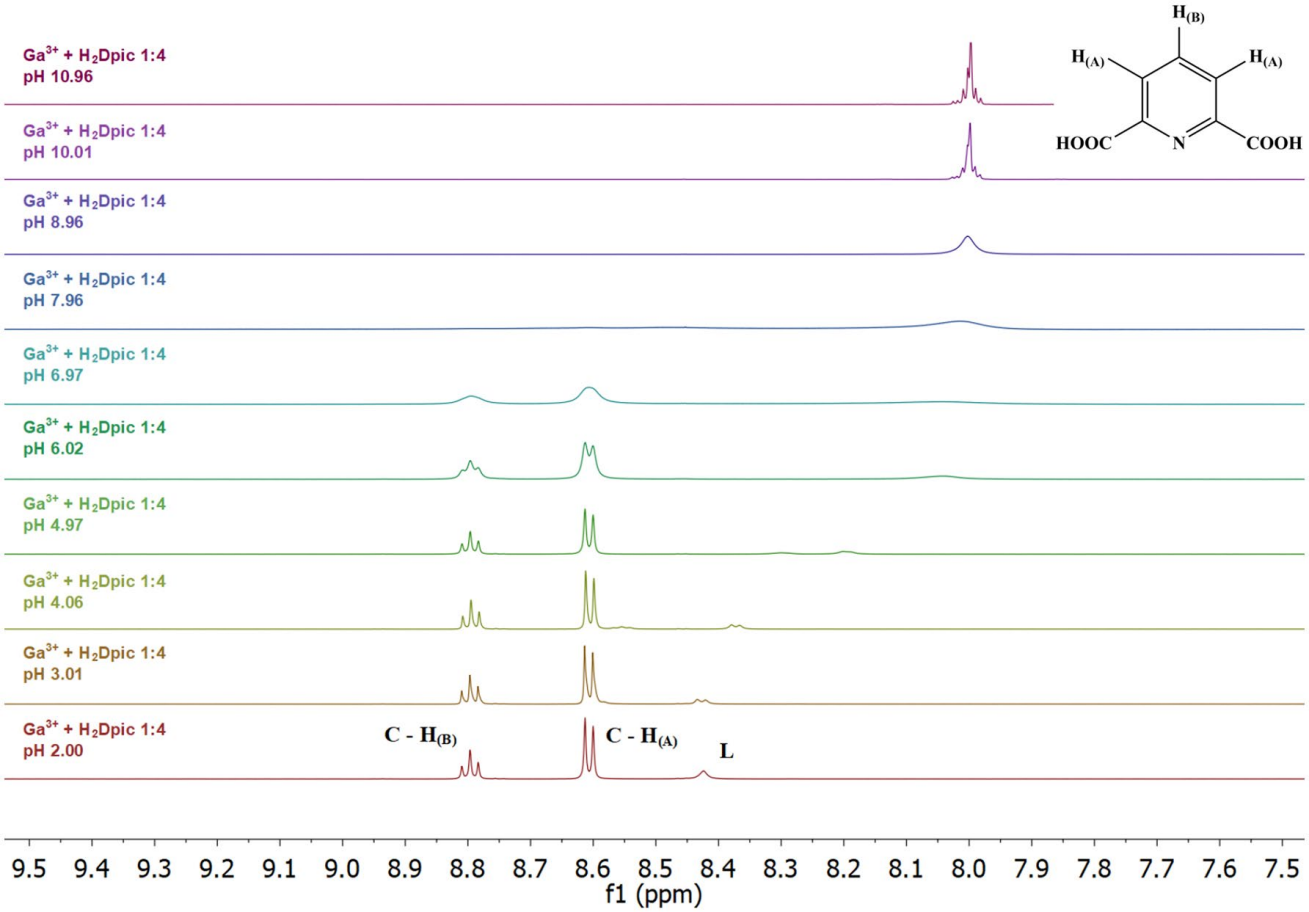
Dependence on pH of the ^1^H NMR spectra of an aqueous solution containing Ga(III) and H_2_Dpic in a 1–4 ratio; c_Ga_ = 8 mM, c_H2Dpic_ = 32 mM

From the ^1^H NMR spectra of Ga(III)-H_2_Dpic system (Fig. 7) we can see significant changes in the position of ligand signals from pH 2 to 7. Considering the results of potentiometric titrations, the observed changes indicate the formation of complex species [Ga(Dpic)_2_]^−^ at pH 2–3 and [Ga(Dpic)_2_(OH)]^2−^ above pH 3. The complex [Ga(Dpic)_2_]^−^ is characterized by ^1^H NMR signals at *δ*_H_ 8.80 (t, *J* 7.8, H_B_) and 8.61 (d, *J* 7.8, H_A_). The ^1^H NMR spectra at pH 6.02 and 6.97 showed broad resonances and this suggested that the pH strongly affects the structure of the complex. The potentiometric titrations showed that two species may coexist in solution: the complex [Ga(Dpic)_2_]^−^ together with complex [Ga(Dpic)_2_(OH)]^2−^. As shown in Fig. 7 the linewidths and intensities of the signals are changing with the pH and composition of the solution. The signals coalesce at pH 7.96 and decoalesce at pH 10.01. In the alkaline region, the complexing species decomposes due to the formation of stable gallium(III) hydroxide species, and therefore only free acid signals in deprotonated Dpic^2−^ form are observed in the solution. In a minority, the presence of free acid can also be observed (at pH 2.00–4.97)., which confirms the position of the signals at *δ*_H_ 8.41 (br s, H_A_, H_B_) at pH 2.00, *δ*_H_ 8.43 (d, *J* 7.8, H_A_) at pH 3.01, *δ*_H_ 8.56 (t, *J* 7.8, H_B_), 8.37 (d, *J* 7.8, H_A_) at pH 4.06, and *δ*_H_ 8.30 (br s, H_B_), 8.20 (br s, H_A_) at pH 4.97.

## Biological tests

### Antimicrobial activity

Table 5 shows the results of antimicrobial activity of gallium(III)complexes and ligands, as well. Ligands HPic, H_2_Chel and H_2_Cldpic did not affect the growth of any model microorganism. Prepared Ga(III) complexes had partially inhibited bacterial growth. We have evaluated the antibacterial activity according to IC_50_ value since we have observed only partial growth inhibition. The inhibition (50%) of *S. aureus* was recorded in the presence of the complex GaChel, GaPic at the concentration of 2 mM. We have also observed partial inhibition (24%) of *S. aureus* growth in the presence of the GaDpic complex (2 mM). The growth of *E. coli* was inhibited only partially; in the presence of GaDpic (30%), GaCldpic (30%) GaChel (9%).

**Table 5:** Antimicrobial activity of Ga(III) complexes, their free ligands and gallium(III) nitrate evaluated by IC_50_ (mM)

	Bacteria			Yeast		*Filamentous fungi*	
	*S. aureus*	*E. coli*	*P. aeruginosa*	*C. parapsilosis*	*R. oryzae*	*A. alternata*	*M. gypseum*
	IC_50_	IC_50_	IC_50_	IC_50_	IC_50_	IC_50_	IC_50_
GaPic	2	> 2	0.05	> 2	> 2	> 2	> 2
GaDpic	> 2	> 2	0.25	> 2	> 2	> 2	> 2
GaChel	2	> 2	0.5	> 2	> 2	> 2	> 2
GaCldpic	> 2	> 2	0.05	> 2	> 2	> 2	> 2
HPic	> 0.5	> 0.5	> 0.5	> 0.5	> 0.5	> 0.5	> 0.5
H_2_Dpic	> 1	> 1	> 1	> 1	0.5	> 1	> 1
H_2_Chel	> 0.5	> 0.5	> 0.5	> 0.5	> 0.5	> 0.5	> 0.5
H_2_Cldpic	> 0.5	> 0.5	> 0.5	> 0.5	> 0.5	> 0.5	> 0.5
Ga(NO_3_)_3_	> 0.5	> 0.5	> 0.5	> 0.5	> 0.5	> 0.5	> 0.5

The most sensitive of the model bacteria to Ga(III) complexes was *P. aeruginosa*. In the presence of some complexes, the growth of *P. aeruginosa* was fully inhibited (100%). The MIC_100_ value was detected in the presence of GaChel at the concentration of 1 mM. We have observed higher inhibition in the presence of GaCldpic and GaPic (MIC = 0.5 mM). Unfortunately, the positive controls represented by cefoxitin (C), ampicillin (A) and piperacillin (P) are significantly more effective under our experimental conditions against the observed bacterial strains (MIC_100_(C) for *S. aureus* = 0.74 µM, MIC_100_(A) for *E. coli* = 2.86 µM, MIC_100_(P) for *P. aeruginosa* = 5.81 µM). The IC_50_ values ​​are listed in the Table 5. The effect of Ga(III) complexes that caused 100% inhibition of bacterial growth was bacteriostatic.

The highest inhibitory effect of tested complexes was achieved on *P. aeruginosa*. *P. aeruginosa* is a major nosocomial pathogen that causes very difficult infection especially in critically ill and immunocompromised patients [59]. *P. aeruginosa* infections are hard to treat because of the multiresistance to clinically used antibiotics. Since iron is essential for bacterial cells to cause infections, depletion of iron or its substitution with other metal such as Ga(III), seems to open a new approach for the antibacterial strategies. Ga(III) cannot be reduced under physiological condition as Fe(III). Finally, it results in inhibition of several iron-dependent biochemical processes. Ga(III) compounds (and GaM among them) have shown antimicrobial potential [60]. These previous studies confirmed that GaM has significant potential in the treatment of several infections, including infections caused by *P. aeruginosa*, similar to our complexes. The tested complexes have some potential for antibacterial treatment and in further research it would be necessary to evaluate the activity of tested Ga(III) complexes in growth media with depleted iron—that would mimic the environment of body fluids (addition of human serum proteins etc.).

## Cytotoxic activity

### Cytotoxic activity of Ga(III) complexes

From the MTS test results given in Table 6 it is clear that free carboxylic acids do not show cytotoxic activity in the studied range (IC_50_ > 200 μM). Similarly, relatively high inhibitory concentrations were observed for Ga(III) complexes against cancer cell lines. Unfortunately, only the GaPic complex shows a higher level of cytotoxic activity against Jurkat, MDA-MB-231 and A2058 cancer cell lines, with only a very slight level of selectivity observed compared to healthy human dermal fibroblasts (HDF). Moreover, compared to the activity of cisPt, our Ga(III) complexes are indeed inactive and non-selective. For comparison, Kaluderovic et al. examined the cytotoxic activity of Ga(III) complexes with different carboxylate ligands against five cancer cell lines (8505C, A253, A549, A2780, DLD-1) [61]. Although the studied free carboxylic acids, similarly as our used acids, did not exhibit any level of cytotoxicity [62], Ga(III) complexes with various carboxylate ligands showed higher cytotoxicity than gallium(III) nitrate, but on the other hand, the cytotoxic effect of cisplatin was several times higher than that of studied complexes against all tested cancer cell lines [61].

**Table 6:** Cytotoxic activity of novel gallium(III) complexes and free ligands (IC_50_: µM)

	HeLa	HCT116	Jurkat	MDA-MB-231	A2058	PaTu 8902	HDF
GaPic	1532.0 ± 108.3	995.4 ± 27.5	175.0 ± 47.7	151.8 ± 74.3	187.4 ± 12.2	271.1 ± 34.3	230.9 ± 23.8
GaDpic	1139.5 ± 135.2	1237.5 ± 58.9	325.0 ± 34.9	307.7 ± 67.8	287.2 ± 59.3	508.8 ± 32.6	288.2 ± 26.6
GaChel	1414.6 ± 155.3	405.7 ± 86.8	417.6 ± 100.3	198.9 ± 45.6	415.0 ± 4.0	610.4 ± 54.0	347.2 ± 25.5
GaCldpic	447.3 ± 134.4	403.7 ± 47.7	405.4 ± 87.0	270.7 ± 68.3	294.0 ± 57.5	437.4 ± 22.6	446.8 ± 27.6
HPic	> 200	> 200	> 200	> 200	> 200	> 200	> 200
H_**2**_**Dpic**	> 200	> 200	> 200	> 200	> 200	> 200	> 200
H_**2**_**Chel**	> 200	> 200	> 200	> 200	> 200	> 200	> 200
H_**2**_**Cldpic**	> 200	> 200	> 200	> 200	> 200	> 200	> 200
cisPt	35.4 ± 3.4	7.4 ± 0.8	6.3 ± 0.5	7.1 ± 0.3	17.6 ± 1.2	12.9 ± 0.9	40.1 ± 2.8

HeLa—human cervical adenocarcinoma, HCT116—human colorectal carcinoma, Jurkat—human leukaemic T cell lymphoma, MDA-MB-231—human mammary gland adenocarcinoma, A2058—human metastatic melanoma, PaTu 8902—human pancreatic adenocarcinoma, HDF—human dermal fibroblasts

On the other hand, the biological activity of gallium(III) complexes is likely to increase by coordination to ligands that themselves exhibit biological activity and thus a significant synergistic effect could be observed. For example, gallium(III) complex [Ga(ClQ)_3_]⋅MeOH (H-ClQ = 5-chloro-8-quinolinol) as well as the ligand itself showed significant cytotoxic activity against tested cancer cell lines (A2780, MDA-MB-231, HCT116), while the activity of the Ga(III) complex was at least 2.5 times higher than the activity of H-ClQ and even higher or comparable to cisplatin activity [63].

### Cytotoxic effect of GaPic and its Ag(I) and Zn(II) analogues against HepG2 cell line

Despite the results of the cytotoxic activity of Ga(III) complexes against different cancer cell lines (see previous section), we decided to test the most effective Ga(III) picolinate complex against HepG2 cells because, as mentioned in the introduction, Ga(III) is well and even specifically accumulated in hepatocellular carcinoma cells but not in non-tumour liver [16, 17, 64].

Our experiments with HepG2 cells cultivated with metal complexes in the concentration range 0.1–25 μM for 24, 48 and 72 h showed only slight decrease of viability of the cells (the results are not given due to their insignificance). This led us to experiments with higher concentrations of metal complexes.

**Fig. 8:**
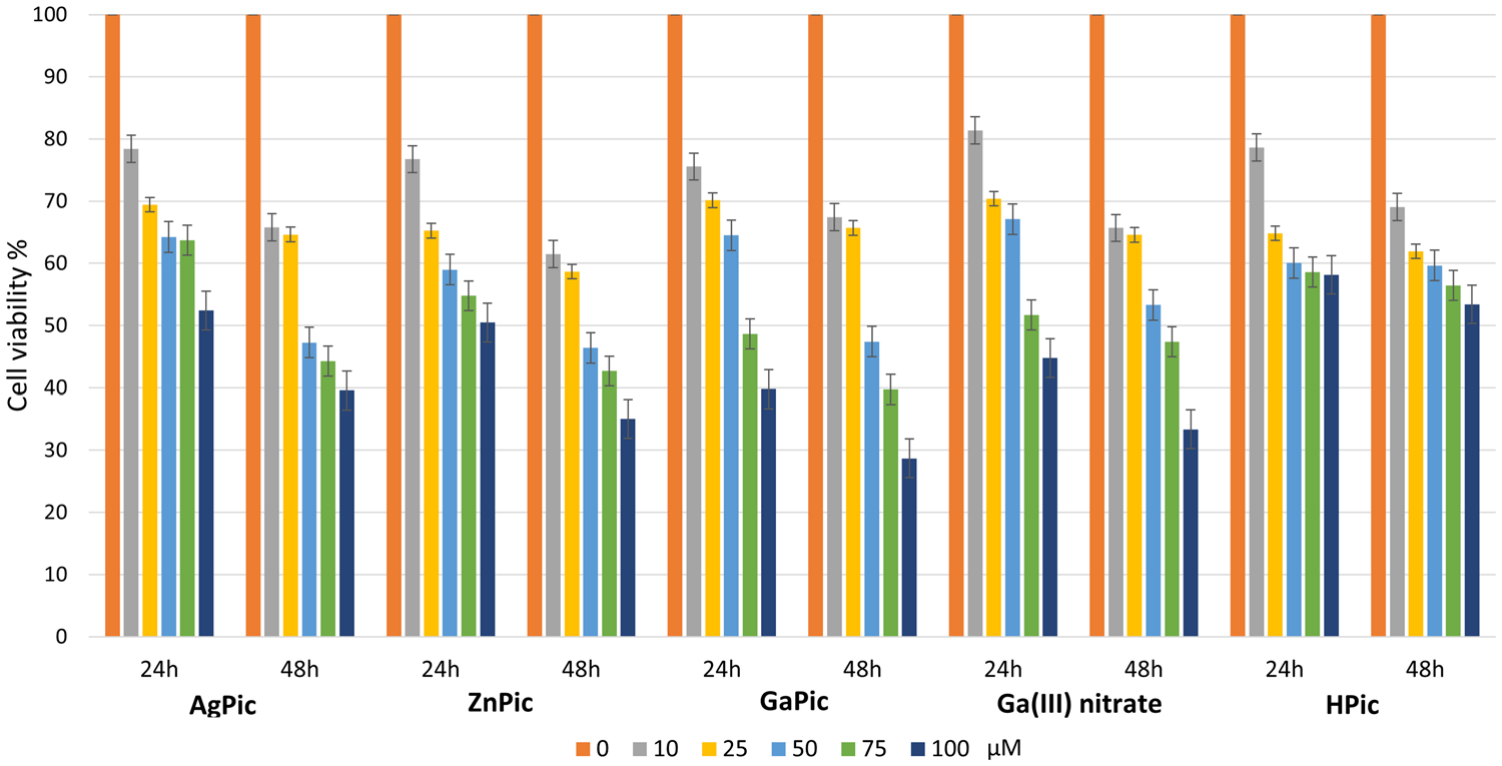
Effect of test compounds on HepG2 cell viability after 24 and 48 h of cultivation. The data represent mean ± standard deviation, n = 3

As can be seen in Fig. 8, even the low concentrations of individual starting materials (gallium(III) nitrate and picolinic acid) influence the HepG2 cancer cells viability. Based on this, a synergic effect can be expected in the case of the GaPic complex. Indeed, more pronounced decrease in the cell viability can be observed due to the GaPic complex at a concentration of 50 μM, but only after 48 h. A similar trend can be observed at higher concentrations (75 and 100 μM).

If we compare the effect of the GaPic complex with its Ag(I) and Zn(II) analogues (previously prepared) [6, 39], we can see that the effect on cell viability after 24 h at lower concentrations (10, 25, 50 μM) is similar for all three complexes. However, the decrease in cell viability is slightly more pronounced for the GaPic complex (40% at 75 μM; 28% at 100 μM) after 48 h than for AgPic (45% at 75 μM; 40% at 100 μM) and ZnPic (42% at 75 μM; 36% at 100 μM).

Comparing our results with the results for GaM, it can be stated that the obtained result is consistent with the published one. While Chua et al. [64] observed the antiproliferative effect of GaM with an IC_50_ concentration of 25–35 μM after 6 days of treatment, we observed a decrease in the viability of HepG2 cells at higher concentrations (75 and 100 μM) but as early as 48 h.

### Plasma protein binding

Albumin is the most abundant carrier protein in the blood plasma of wide variety of organisms and plays a principal function in the regulation of its osmotic pressure and the binding and transportation of various endo- and exogenous compounds such as fatty acids and drugs [65]. Plasma protein binding is a significant factor to understand the pharmacokinetic and pharmacodynamic properties of drugs, as it effectively influences drug distribution and determines the free fraction, which is available to the target [66].

**Fig. 9:**
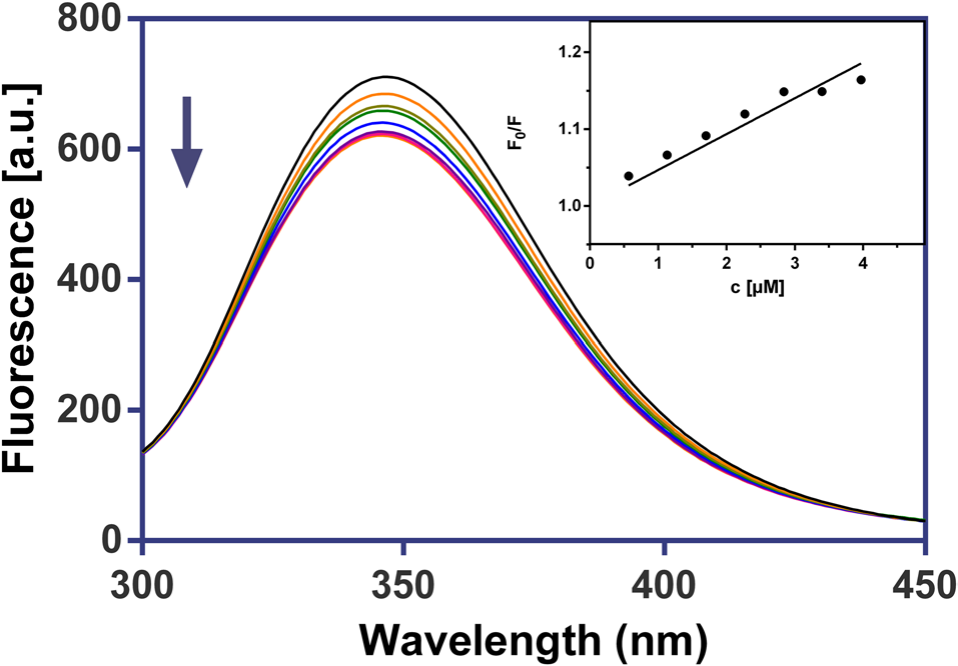
Fluorescence emission spectra of BSA in the presence of GaPic (0–4 μM), in 10 mM

**Fig. 10:**
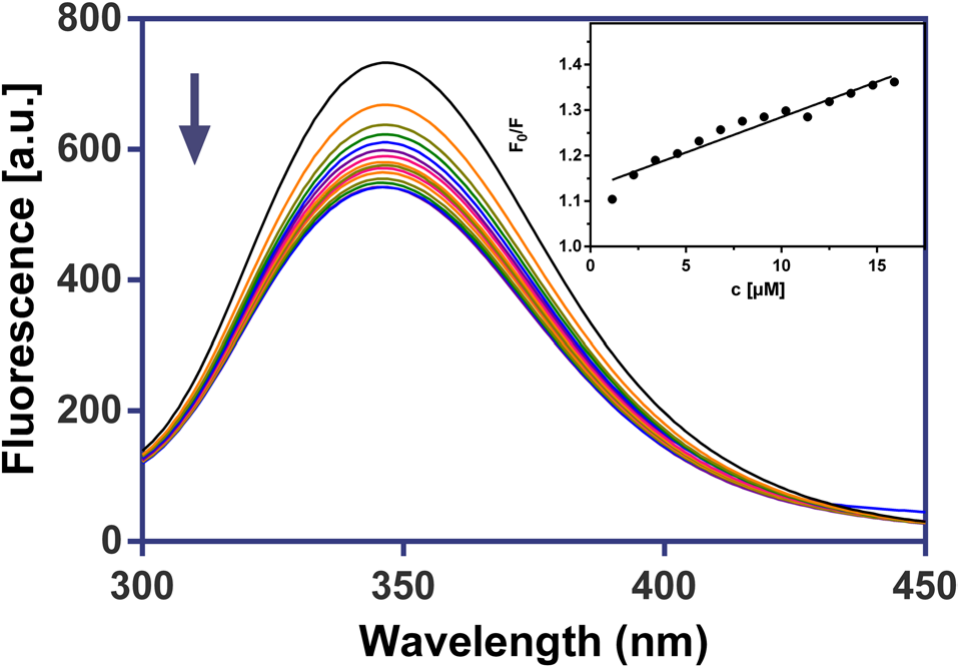
Fluorescence emission spectra of BSA in the presence of GaDpic (0–15.9 µM), in 10 mM PBS buffer (λ_ex_ = 280 nm, pH 7.4, 25 °C). Inset: the corresponding Stern–Volmer plot

We have utilised fluorescence quenching technique to study the interaction between Ga(III) complexes and bovine serum albumin (BSA). The fluorescence spectra of BSA with investigated complexes and the ligands themselves are shown in Figs. 9 and 10, S7 and S8.

We have observed that the fluorescence intensity of BSA decreased regularly with an increased concentration of complexes or ligands. The *K*_*SV*_ constants for complex-BSA system show values in the order of 10^4^ M^−1^ (Table 7), which suggest the significant binding affinity of investigated drugs to important plasma protein.

**Table 7:** Stern–Volmer binding constants (*K*_*sv*_) for the interactions of ligands HPic, H_2_Dpic and Ga(III) complexes with BSA

*K*_*sv*_^*BSA*^	HPic	H_2_Dpic	GaPic	GaDpic
**[**M^−1^**]**	0.68 × 10^4^	2.87 × 10^4^	4.67 × 10^4^	6.85 × 10^4^

The higher value of the *K*_*SV*_ constant (6.85 × 10^4^ M^−1^) was found for the GaDpic complex. Our found Stern–Volmer constants are similar to those calculated for silver(I) complex with 1,8-naphthyridine ligand by Ašanin et al. [67].

PBS buffer (λex = 280 nm, pH 7.4, 25 °C). Inset: the corresponding Stern–Volmer plot.

## Conclusion

Simple one-step crystallization was used for novel GaPic, GaDpic, GaChel and GaCldpic complexes preparation with the aim to evaluate their structural and biological properties. X-ray, elemental, mid-IR and thermal analyses completely characterized the complexes in terms of their composition and structure and pointed out the significant influence of binding and intermolecular interactions on their spectral and thermal properties.

Just as picolinic acid takes its typical *N,O*-bidentate chelating coordination mode, dipicolinic acid and its -Cl and -OH derivatives coordinate to the gallium(III) central atom via tridentate chelating *O,N,O*-binding. Moreover, Ga(III) distorted octahedral geometry is completed by two oxygen atoms from two hydroxide-bridging ligands and by one atom from aqua ligand in the case of GaChel and GaCldpic complexes. The results of mid-IR spectroscopy correlate with structural and thermal analyses results. While GaPic, GaDpic and GaChel dehydrate (up to 200 °C) before decomposition, GaCldpic as an anhydrous complex starts to decompose above 200 °C. In addition, Ga-HPic and Ga-H_2_Dpic systems speciation solution study confirms presence of the stable complex species in the pH range from acidic to neutral. To confirm the complexes stability in 1% DMSO (primary solvent for biological testing), time-scale ^1^H NMR spectra were measured (immediately after dissolution up to 96 h). More significant antimicrobial activity of the prepared complexes was observed only against difficult to treat and multidrug-resistant *P. aeruginosa* and anticancer effect only of GaPic complex against Jurkat, MDA-MB-231 and A2058 cancer cell lines. Moreover, the decrease in cell viability of HepG2 cancer cell line at 75 and 100 μM concentrations was observed in a relatively short time (up to 48 h) compared to GaM analogue (25–35 μM, up to 6 days) [64]. In addition, GaPic, GaDpic complexes as well as their ligands quench BSA emission, which suggests good binding to this plasma protein.

In accordance with our results and their comparison with published ones, we can conclude that in order for Ga(III)—pyridine carboxylates to approach the potential therapeutic activity of 2nd generation Ga(III) complexes (GaM and KP46), it would be necessary to evaluate the activity of the tested Ga(III) complexes in iron-depleted growth media and increase their bioavailability.

Unfortunately, based on our time-consuming actual experiments, we can finally answer the question from the manuscript title: Ga(III) pyridinecarboxylate complexes do not have such a biological effect as would be expected based on the structural similarity to GaM and KP46.

## Supporting information

The supporting information is available free of charge at details of crystal data and structure refinement, parameters of hydrogen bonds and π–π interactions, variable pH ^1^H NMR spectra, IR spectra, TG curves, time-dependent ^1^H NMR spectra, fluorescence spectra.

## Accession codes

CCDC 2183166—2,183,169 contain the supplementary crystallographic data for this paper. These data can be obtained free of charge via www.ccdc.cam.ac.uk/data_request/cif, or by emailing data_request@ccdc.cam.ac.uk, or by contacting The Cambridge Crystallographic Data Centre, 12 Union Road, Cambridge CB2 1EZ, UK; fax: + 44 1223 336,033.

## Supplementary Information

Below is the link to the electronic supplementary material.

Supplementary file1 (PDF 841 KB)
