# Supplementary material for: Ga(III) pyridinecarboxylate complexes: potential analogues of the second generation of therapeutic Ga(III) complexes?
Source: J Biol Inorg Chem. 2023 Jul 27;28(6):591–611. doi: 10.1007/s00775-023-02012-2 (PMC10415494; doi:10.1007/s00775-023-02012-2)
Supplement: Supplementary file 1 — Supplementary file1 (PDF 841 KB) [file 775_2023_2012_MOESM1_ESM.pdf]

Supplementary material

Ga(III) pyridinecarboxylate complexes – potential analogues of the second generation of therapeutic Ga(III) complexes?

Michaela Rendošová[1]*, Róbert Gyepes[2], Simona Sovová[3], Danica Sabolová[3], Mária Vilková[4], Petra Olejníková[5], Martin Kello[6], Boris Lakatoš[5], Zuzana Vargová[1]*

[1]*Department of Inorganic Chemistry, P. J. Šafárik University, Moyzesova 11, 041 54 Košice, Slovak Republic*

[2]*Department of Inorganic Chemistry, Charles University, Hlavova 2030, 128 00 Prague, Czech Republic*

[3]*Department of Biochemistry, P. J. Šafárik University, Moyzesova 11, 041 54 Košice, Slovak Republic*

[4]*NMR Laboratory, P. J. Šafárik University, Moyzesova 11, 041 54 Košice, Slovak Republic*

[5]*Department of Biochemistry and Microbiology, Slovak University of Technology, Radlinského 9, 812 37 Bratislava, Slovak Republic*

[6]*Department of Pharmacology, P. J. Šafárik University, Trieda SNP 1, 040 11 Košice, Slovak Republic*

Abstract

A series of novel Ga(III) – pyridinecarboxylates ([Ga(Pic)$_3$]·H_2O (GaPic; HPic = picolinic acid), H_3O[Ga(Dpic)$_2$]·H_2O (GaDpic; H_2Dpic = dipicolinic acid), [Ga(Chel)(H_2O)(OH)]$_2$·4H_2O (GaChel; H_2Chel = chelidamic acid) and [Ga(Cldpic)(H_2O)(OH)]$_2$ (GaCldpic; H_2Cldpic = 4-chlorodipicolinic acid)) have been synthesized by simple one-step procedure. Vibrational spectroscopy (mid-IR), elemental analysis, thermogravimetric analysis and X-ray diffraction confirmed complexes molecular structure, inter and intramolecular interactions and their influence to spectral and thermal properties. Moreover, complex species speciation was described in Ga(III)-HPic and Ga(III)-H_2Dpic systems by potentiometry and ^{1}H NMR spectroscopy and mononuclear complex species were determined; [Ga(Pic)$_2$]$^+$ (logβ_{021} = 16.23(6)), [Ga(Pic)$_3$] (logβ_{031} = 20.86(2)), [Ga(Dpic)$_2$]$^-$ (logβ_{021} = 15.42(9)) and [Ga(Dpic)$_2$(OH)]$^{2-}$ (logβ_{-121} = 11.08(4)). To confirm the complexes stability in 1% DMSO (primary solvent for biological testing), time-scale ^{1}H NMR spectra were measured (immediately after dissolution up to 96 hours). Antimicrobial activity evaluated by IC_{50} (0.05mM) is significant for GaDpic and GaCldpic against difficult to treat and multiresistant *P. aeruginosa*. On the other hand, the GaPic complex is most effective against Jurkat, MDA-MB-231 and A2058 cancer cell lines and significantly also decreases the HepG2 cancer cells viability at 75 and 100 µM concentrations in a relatively short time (up to 48 hours). In addition, fluorescence measurements have been used to elucidate BSA binding activity between ligands, Ga(III) complexes and bovine serum albumin.

Table S1. Crystal data and structure refinement for gallium(III) complexes.

	**GaPic**	**GaDpic**	**GaChel**	**GaCldpic**
Empirical formula	$C_{18}H_{14}Ga_1N_3O_7$	$C_{14}H_{11}Ga_1N_2O_{10}$	$C_{14}H_{20}Ga_2N_2O_{18}$	$C_{14}H_{10}Cl_2Ga_2N_2O_{12}$
Formula weight	454.04	436.97	643.76	608.58
Temperature [K]	150(2)	120(2)	120(2)	120(2)
Wavelength [Å]	0.71073	0.71073	1.54178	0.71073
Crystal system	Monoclinic	Orthorhombic	Triclinic	Orthorhombic
Space group	*C*2/*c*	*Pna*2	*P*-1	*Pbca*
Unit cell dimensions	a = 30.0494(9)	a = 8.9170(4)	a = 6.8748(2)	a = 9.3343(5)
[Å, °]	b = 8.4813(3)	b = 16.0726(7)	b = 7.9045(2)	b = 10.0858(5)
	c = 13.8890(4)	c = 10.8946(4)	c = 10.5559(3)	c = 20.4124(9)
	α = 90	α = 90	α = 80.7300(10)	α = 90
	β = 95.5370(10)	β = 90	β = 74.9970(10)	β = 90
	γ = 90	γ = 90	γ = 67.0720(10)	γ = 90
Volume [$Å^3$]	3523.21(19)	1561.41(11)	509.13(2)	1921.70(16)
Z	8	4	1	4
Calculated density [$g.cm^{-3}$]	1.712	1.859	2.100	2.103
Absorption coefficient [mm^{-1}]	1.613	1.827	4.217	3.154
F(000)	1840	880	324	1200
Crystal size [mm^3]	0.386 x 0.359 x 0.096	0.282 x 0.246 x 0.174	0.168 x 0.107 x 0.103	0.676 x 0.506 x 0.395
θ range for data collection [°]	2.496 – 27.511	2.258 – 28.047	4.346 – 70.095	3.582 – 27.509
Index ranges	$-38\leq h\leq 32$, $-11\leq k\leq 10$, $-18\leq l\leq 12$	$-11\leq h\leq 11$, $-21\leq k\leq 21$, $-14\leq l\leq 14$	$-8\leq h\leq 8$, $-9\leq k\leq 9$, $-11\leq l\leq 12$	$-11\leq h\leq 12$, $-13\leq k\leq 13$, $-26\leq l\leq 26$
Reflections collected/unique	27567 / 3999	30503 / 3778	8669 / 1913	43219 / 2194
Data / restraints / parameters	3999 / 0 / 270	3778 / 1 / 266	1913 / 1 / 195	2194 / 0 / 158
Goodness-of-fit on F^2	1.029	1.084	1.114	1.131
Final R indices [$I > 2\sigma(I)$]	$R1$ = 0.0255; $wR2$ = 0.0569	$R1$ = 0.0182; $wR2$ = 0.0449	$R1$ = 0.0247; $wR2$ = 0.0669	$R1$ = 0.0183; $wR2$ = 0.0482
R indices (all data)	$R1$ = 0.0352; $wR2$ = 0.0600	$R1$ = 0.0201; $wR2$ = 0.0458	$R1$ = 0.0258; $wR2$ = 0.0677	$R1$ = 0.0190; $wR2$ = 0.0486
Largest diff. peak and hole [$e.Å^{-3}$]	0.321, -0.264	0.292, -0.350	0.594, -0.523	0.472, -0.295

Table S2. IR spectral data assignments for gallium(III) complexes and their appropriate ligands.

	**HPic**	**GaPic**	**H_2Dpic**	**GaDpic**	**H_2Chel**	**GaChel**	**H_2Cldpic**	**GaCldpic**
ν(OH)	-	3527 m	-	3463 m, br	3603 m	3615 m	3481 m	3225 m,br
		3451 m			3442 m	3440 m, br	3377 m	
ν(CH)	3112 m	3111 w	3067 w	3054 m	3080 w	3067 m	3092 w	3076 m
	3053 w	3061 w						
ν(C=O)	1708 m	-	1697 s	-	1716 m	-	1727 s	-
$\delta(H_2O)$	-	1639 m		1601 m		1587 m	-	1601 m
$\nu_{as}(COO^-)$	-	1670 s	-	1635 s		1656 m	-	1702 s
								1673 s
$\nu_s(COO^-)$	-	1333 s		1363 s		1392 s		1354 s
ν(CC)+	1592 m	1606 m	1574 m	1581 m	1609 s	1613 s	1577 m	1579 m
ν(CN)	1571 m	1569 m	1455 m	1440 m	1568 w, sh	1558 w	1478 w	1455 w
	1526 m	1471 m			1470 m	1470 w	1439 m	1437 m
	1453 m	1452 m						
β(CCH)	1293 m	1291 m	1296 m	1287 m	1258 m	1242 m	1212 m	1201 m
	1157 m	1162 m	1161 m	1149 m	1132 m	1136 m	1172 s	1111 m
	1082 m	1069 m	1079 m	1083 s				
$\beta_{\text{ring } \textit{breathing}}$	1008 m	1029 m	995 m	1043 m	1021 m	1069 s	998 m	1047 m
γ(CCH)	749 s	760s	748 s	776 s	760 m	761 m	731 m	752 s
	678 s	693 s	647 s	684 s				

Abbreviation: band intensity s – strong, m – medium, w – weak, sh – shoulder; br – broad; type of vibrations: ν – stretching, β – in-plane bending, γ – out-of-plane bending.

Table S3. Possible hydrogen bonds for GaPic.

**D–H···A**	***d*(D–H) [Å]**	***d*(H...A) [Å]**	***d*(D...A) [Å]**	**<(DHA) [°]**
O7–H7A···O1^{i}	0.94(3)	1.94(3)	2.871(2)	169(2)
O7–H7B···O6^{ii}	1.00(4)	1.83(4)	2.814(2)	170(3)
C3–H3···O7	0.95	2.21	3.0902	154
C4–H4···O2^{i}	0.95	2.54	3.4713	167
C9–H9···O2^{iii}	0.95	2.40	3.1632	137
C10–H10···O1^{iv}	0.95	2.59	3.4942	160
C15–H15···O6^{iv}	0.95	2.33	3.1422	143
C18-H18···O4^{v}	0.95	2.46	3.2072	135

Symmetry transformation used to generate equivalent atoms: (i) x, 1+y, z; (ii) x, 1-y, -1/2+z; (iii) -x, -y, -z; (iv) x, -y, -1/2+z; (v) 1/2-x, -1/2+y, 1/2-z.

Table S4. Cg···Cg distances and angles characterizing π–π interactions in GaPic.

**Cg(I)···Cg(J)**	***d*(Cg(*I*)···Cg(*J*)) [Å]**	**α [°]**	**β [°]**	**γ [°]**
Cg1···Cg1^{i}	3.6425(10)	2.55(8)	17.6	17.6
Cg1···Cg1^{ii}	3.8763(10)	0.00(8)	27.3	27.3

Symmetry transformation used to generate equivalent atoms: (i) -x, y, 1/2-z; (ii) -x, -y, -z.

α is the dihedral angle between planes I and *J*; *β* is the angle between Cg(*I*)···Cg(*J*) vector and normal to plane *I*; *γ* is the angle between Cg(*I*)···Cg(*J*) vector and normal to plane *J*.

Table S5. Possible hydrogen bonds for GaDpic.

**D–H···A**	***d*(D–H) [Å]**	***d*(H...A) [Å]**	***d*(D...A) [Å]**	**<(DHA) [°]**
O9–H9A···O10^{i}	0.96(6)	1.52(6)	2.461(3)	165(5)
O9–H9B···O6	0.79(3)	1.86(4)	2.644(3)	171(4)
O9–H9C···O2^{ii}	0.97(5)	1.58(5)	2.548(3)	173(4)
O10–H10A···O4^{iii}	0.76(5)	1.92(5)	2.645(3)	160(5)
O10–H10B···O8^{iv}	0.99(5)	1.71(5)	2.682(3)	168(4)
C5–H5···O8^{v}	0.95	2.36	3.209(3)	148
C7–H7···O6^{vi}	0.95	2.29	3.173(3)	154
C14–H14···O2^{vii}	0.95	2.48	3.350(3)	152

Symmetry transformation used to generate equivalent atoms: (i) 1-x, -y, -1/2+z; (ii) 1-x, -y, 1/2+z; (iii) -x, -y, 1/2+z; (iv) 1+x, y, 1+z; (v) -1/2-x, -1/2+y, 1/2+z; (vi) 1/2-x, -1/2+y, -1/2+z; (vii) 1/2-x, 1/2+y, 1/2+z.

Table S6. Possible hydrogen bonds for GaChel.

**D–H···A**	***d*(D–H) [Å]**	***d*(H...A) [Å]**	***d*(D...A) [Å]**	**<(DHA) [°]**
O5–H5A···O2^{i}	0.78(3)	1.93(4)	2.702(2)	173(4)
O5–H5B···O9	0.79(4)	1.81(4)	2.592(3)	175(4)
O6–H6···O2^{ii}	0.76(3)	2.03(3)	2.782(2)	171(3)
O7–H7···O4^{iii}	0.79(4)	1.91(4)	2.674(2)	164(3)
O8–H8A···O5^{i}	0.76(4)	2.52(4)	3.129(3)	139(3)
O8–H8B···O1	0.89(3)	1.98(3)	2.859(2)	169(3)
O9–H9A···O8^{iv}	0.74(4)	2.18(4)	2.903(3)	164(4)
O9–H9B···O4^{v}	0.78(3)	1.97(3)	2.735(2)	167(3)
C2–H2···O3^{iii}	0.95	2.21	3.122(3)	160
C4–H4···O8^{vi}	0.95	2.49	3.341(3)	148

Symmetry transformation used to generate equivalent atoms: (i) -x, 1-y, 1-z; (ii) x, -1+y, z; (iii) x, 1+y, z; (iv) -x, -y, 1-z; (v) 1-x, -y, -z; (vi) 1+x, y, -1+z.

Table S7. Possible hydrogen bonds for GaCldpic.

**D–H···A**	***d*(D–H) [Å]**	***d*(H...A) [Å]**	***d*(D...A) [Å]**	**<(DHA) [°]**
O5–H5A···O1^{i}	0.80(2)	1.90(2)	2.6639(15)	162.5(19)
O5–H5B···O3^{ii}	0.79(3)	1.88(3)	2.6530(16)	169(3)
O6–H6···O4^{ii}	0.73(3)	2.00(3)	2.7283(15)	176(3)
C2–H2···O4^{iii}	0.95	2.50	3.3719(18)	153
C4–H4···O2^{iv}	0.95	2.36	3.2694(18)	161

Symmetry transformation used to generate equivalent atoms: (i) 1/2-x, -1/2+y, z; (ii) 1/2+x, 1/2-y, -z; (iii) -x, 1/2+y, 1/2-z; (iv) -x, -1/2+y, 1/2-z.

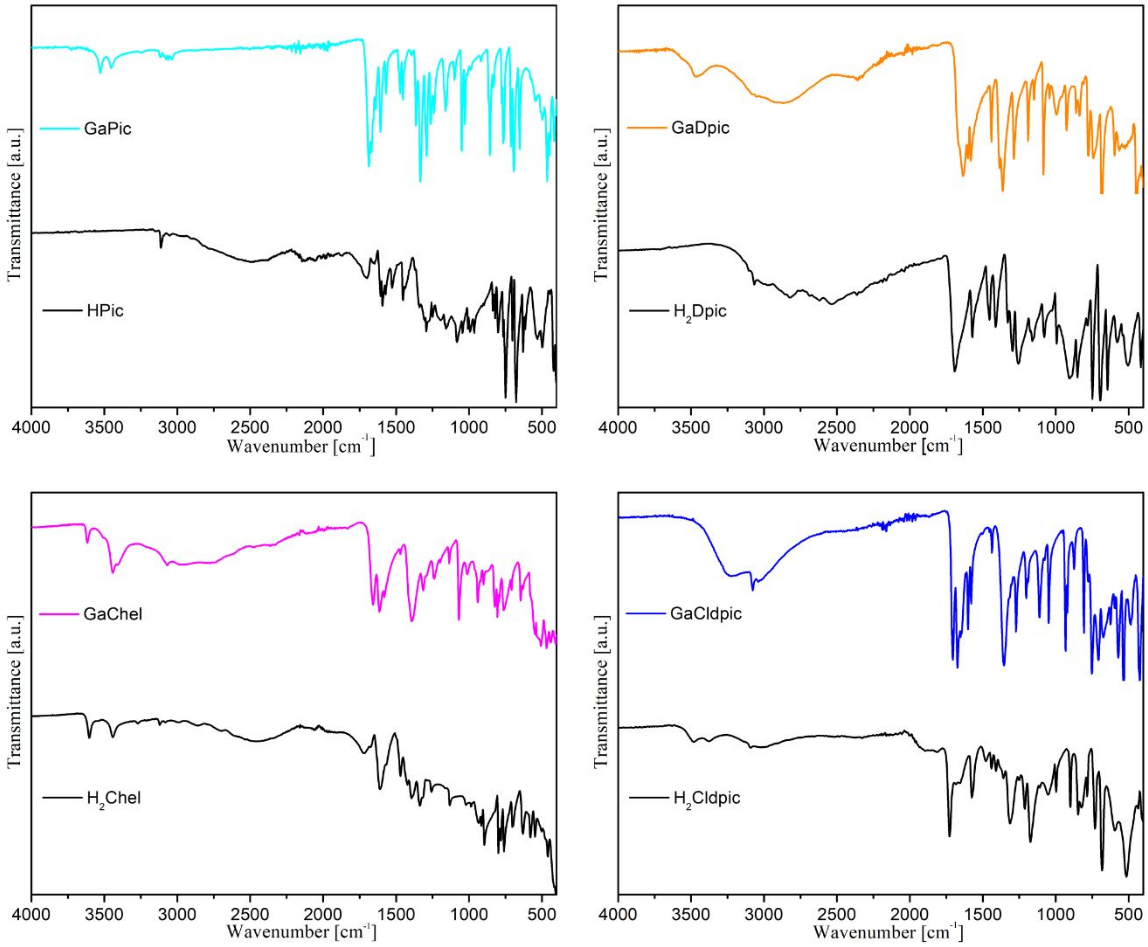

Fig. S1. IR spectra of gallium(III) complexes and their appropriate ligands.

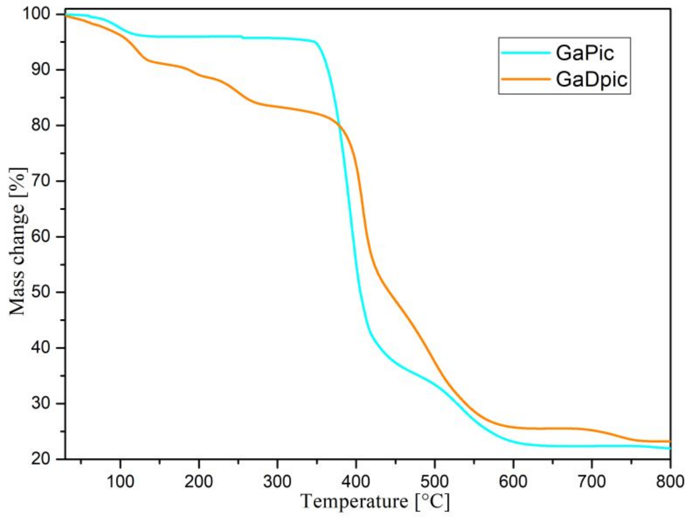

Fig. S2. Thermogravimetric curves of prepared gallium(III) complexes GaPic and GaDpic measured in air atmosphere.

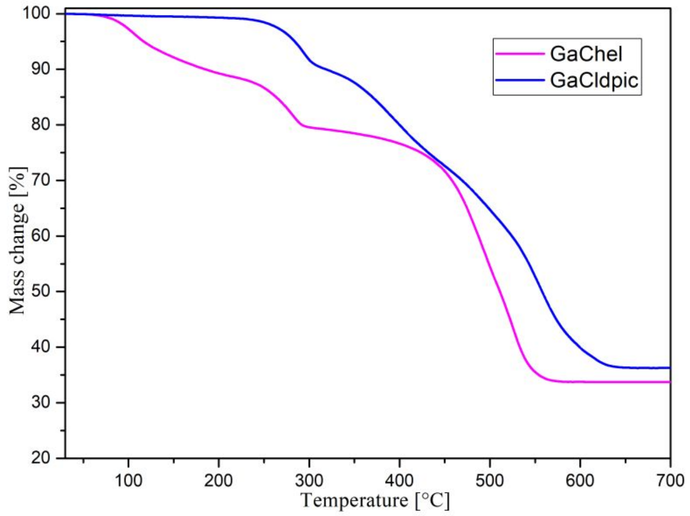

Fig. S3. Thermogravimetric curves of prepared gallium(III) complexes GaChel and GaCldpic measured in air atmosphere.

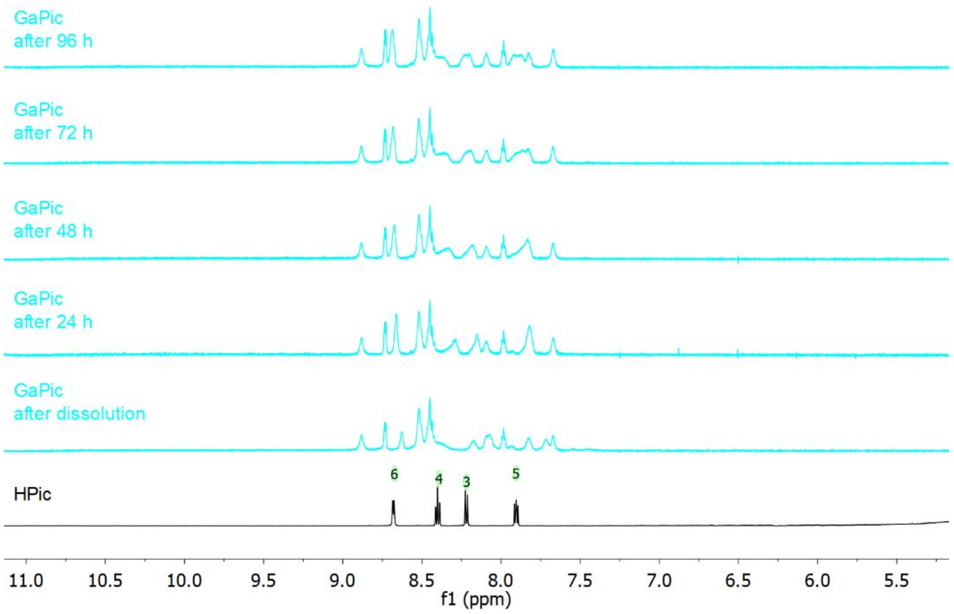

Fig. S4A. ^{1}H NMR spectra (600 MHz) of HPic ligand (δ_H 8.68 (1H, d, J = 5.4 Hz, H-6), 8.40 (1H, td, J = 7.9, 1.6 Hz, H-4), 8.22 (1H, dd, J = 7.9, 1.2 Hz, H-3), 7.90 (ddt, J = 7.6, 5.4, 1.2 Hz, H-5)) and time-dependent ^{1}H NMR spectra (600 MHz) of GaPic complex (δ_H 8.88 (1H, br s), 8.73 (1H, d, J = 5.33 Hz, H), 8.63 (1H, br s), 8.52 (3H, br s), 8.45 (2H, m), 8.17 (1H br s), 8.08 (1H, m), 7.99 (1H, t, J = 6.3 Hz), 7.83 (1H, br s), 7.72 (1H, br s), 7.67 (1H, br s)) ppm, recorded in 1 % DMSO-d_6/D_2O. pH of ligand solution was adjusted to the pH value of complex solution (pH = 5.1).

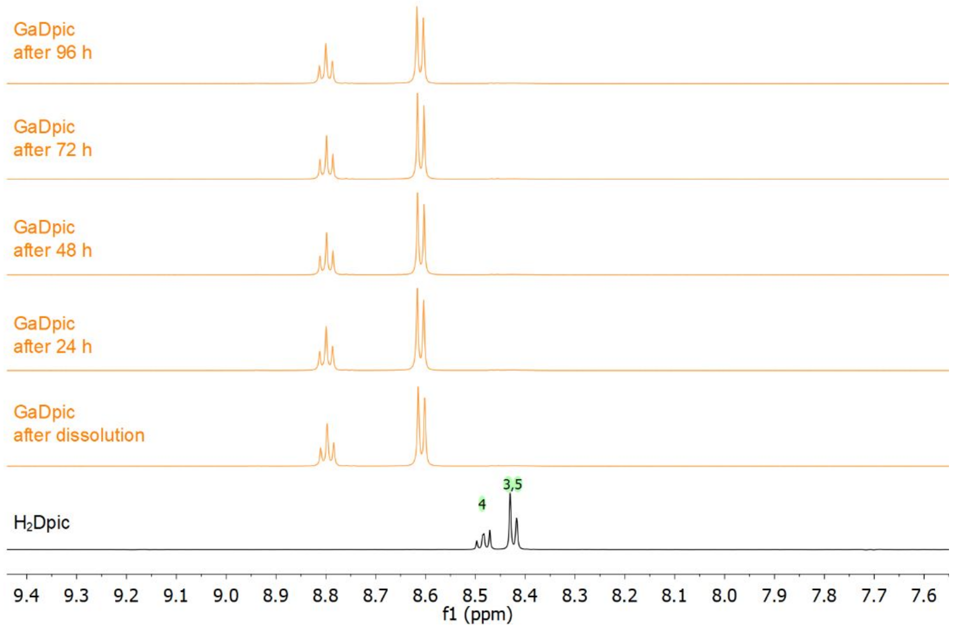

Fig. S4B. ^{1}H NMR spectra (600 MHz) of H_2Dpic ligand (δ_H 8.48 (1H, dd, J = 8.6, 6.9 Hz, H-4), 8.42 (2H, d, J = 7.7 Hz, H-3,5)) and time-dependent ^{1}H NMR spectra (600 MHz) of GaDpic complex (δ_H 8.80 (1H, t, J = 7.8 Hz, H-4), 8.61 (2H, d, J = 7.8 Hz, H-3,5)) ppm, recorded in 1 % DMSO-d_6/D_2O. pH of ligand solution was adjusted to the pH value of complex solution (pH = 2.1).

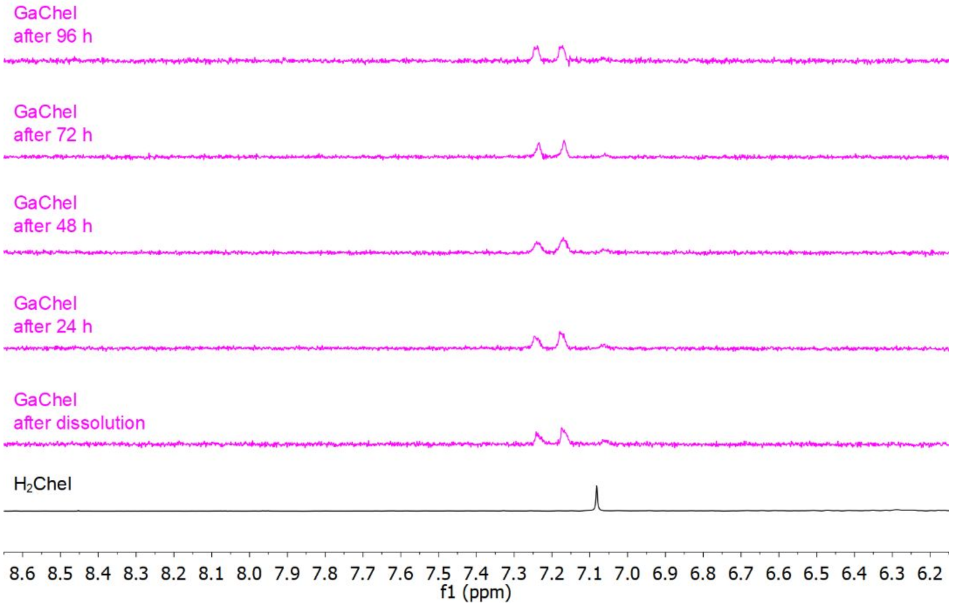

Fig. S4C. ^{1}H NMR spectra (600 MHz) of H_2Chel ligand (δ_H 7.08 (s, 2H)) and time-dependent ^{1}H NMR spectra (600 MHz) of GaChel complex (δ_H 7.24 (1H, br s), 7.17 (1H, br s)) ppm, recorded in 1 % DMSO-d_6/D_2O. pH of ligand solution was adjusted to the pH value of complex solution (pH = 4.9).

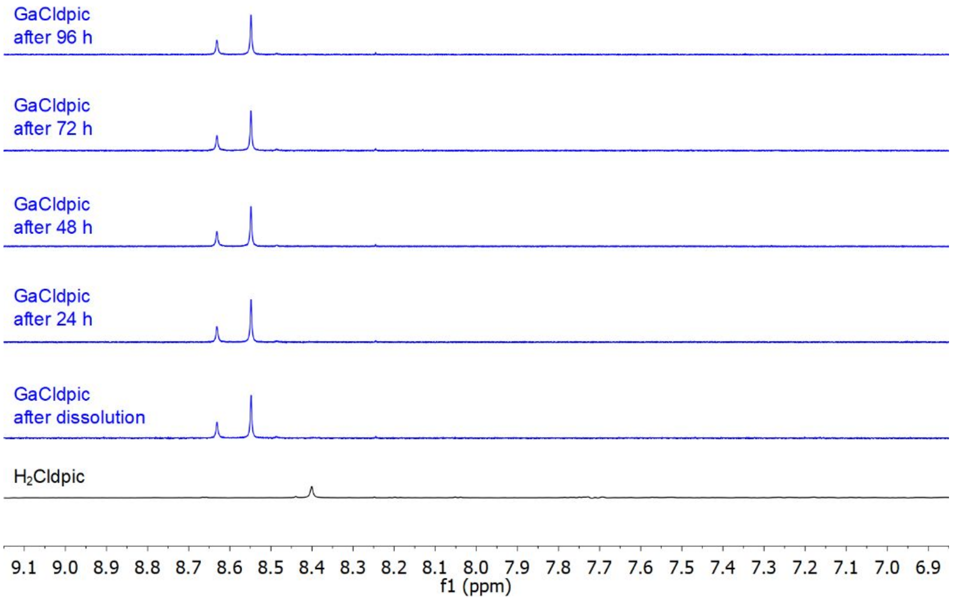

Fig. S4D. ^{1}H NMR spectra (600 MHz) of H_2Cldpic ligand (δ_H 8.40 (s, 2H)) and time-dependent ^{1}H NMR spectra (600 MHz) of GaCldpic complex (δ_H 8.63 (1H, br s), 8.55 (2H, br s)) ppm, recorded in 1 % DMSO-d_6/D_2O. pH of ligand solution was adjusted to the pH value of complex solution (pH = 2.1).

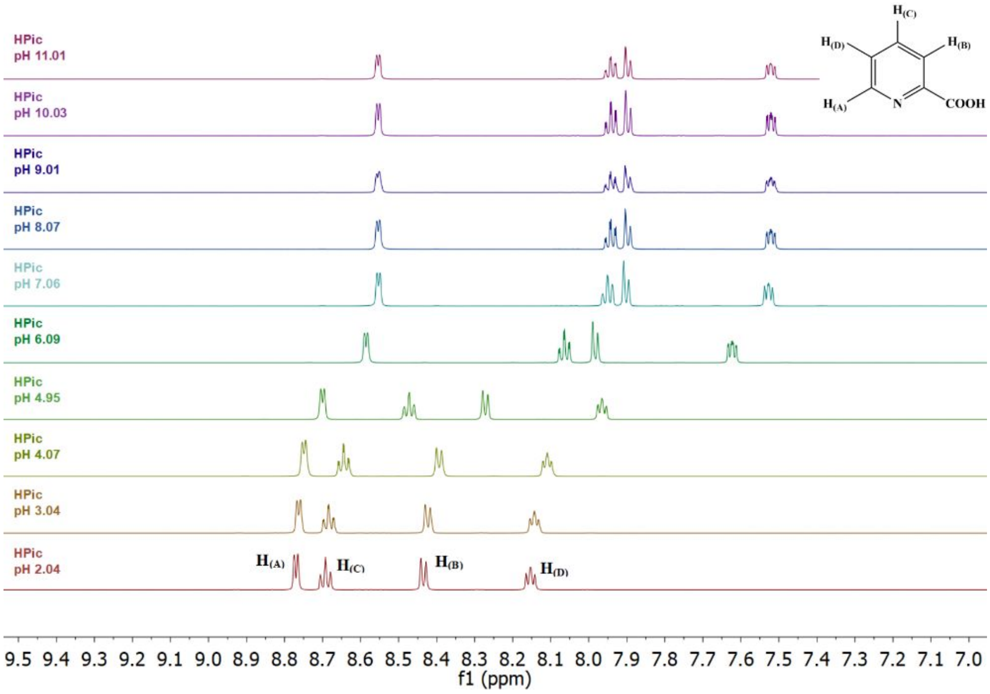

Fig. S5. Dependence on pH of the ^{1}H NMR spectra of HPic aqueous solution.

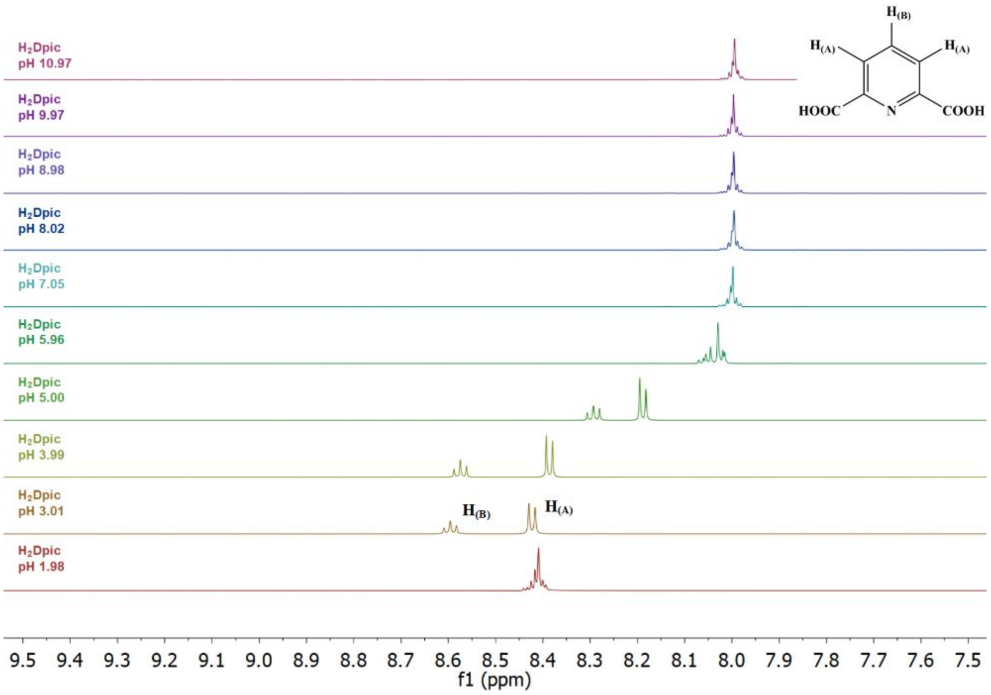

Fig. S6. Dependence on pH of the ^{1}H NMR spectra of H_2Dpic aqueous solution.

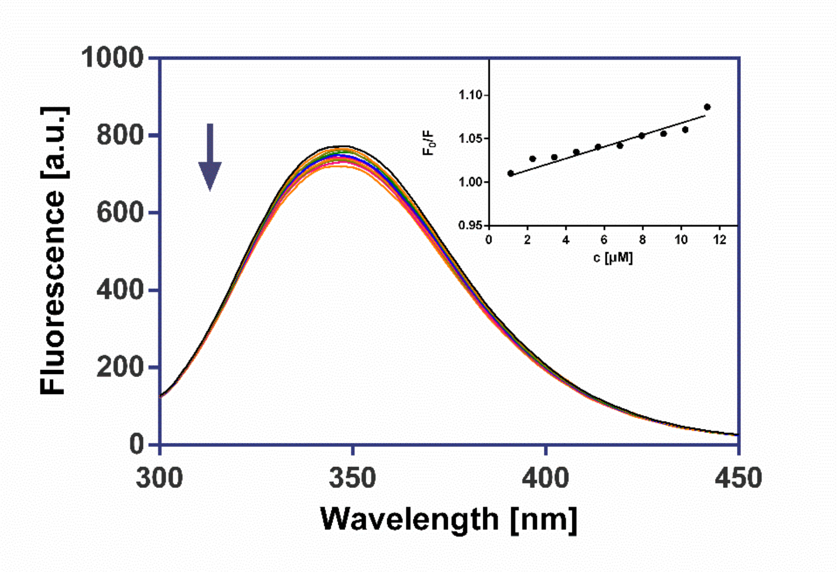

Fig. S7. Fluorescence emission spectra of BSA in the presence of Pic (0 –11.4 µM), in 10 mM PBS buffer (λ_{ex}=280nm, pH 7.4, 25 °C). Inset: the corresponding Stern-Volmer plot.

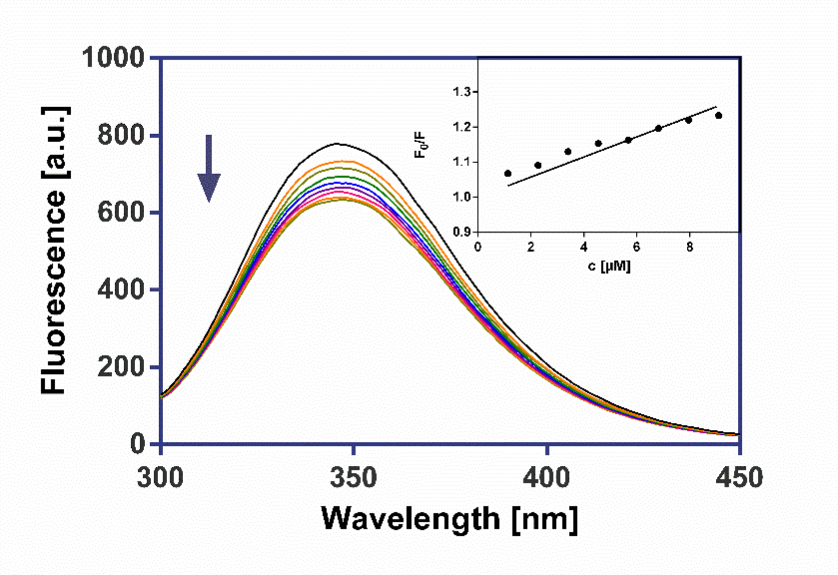

Fig. S8. Fluorescence emission spectra of BSA in the presence of H_2Dpic (0 –9.1 μM), in 10 mM PBS buffer (λ_{ex}=280nm, pH 7.4, 25 °C). Inset: the corresponding Stern-Volmer plot.
